# What Works to Improve Wellbeing? A Rapid Systematic Review of 223 Interventions Evaluated with the Warwick-Edinburgh Mental Well-Being Scales

**DOI:** 10.3390/ijerph192315845

**Published:** 2022-11-28

**Authors:** Joanna M. Blodgett, Jack M. Birch, Margherita Musella, Frances Harkness, Aradhna Kaushal

**Affiliations:** 1 Kohlrabi Consulting, Manchester SK4 3HJ, UK; 2 Institute of Sport Exercise & Health, Division of Surgery & Interventional Science, University College London, London W1T 7HA, UK; 3 Homerton College, University of Cambridge, Cambridge CB2 8PH, UK; 4 What Works Centre for Wellbeing, London SW1H 9EA, UK; 5 Institute of Epidemiology and Health Care, University College London, London WC1E 7HB, UK

**Keywords:** wellbeing, evaluation, intervention, rapid review, Warwick-Edinburgh Mental Wellbeing Scale

## Abstract

Introduction: The Warwick-Edinburgh Mental Wellbeing Scale (WEMWBS) is a commonly used scale of mental wellbeing focusing entirely on the positive aspect of mental health. It has been widely used in a broad range of clinical and research settings, including to evaluate if interventions, programmes or pilots improve wellbeing. We aimed to systematically review all interventions that used WEMWBS and evaluate which interventions are the most effective at improving wellbeing. Methods: Eligible populations included children and adults, with no health or age restrictions. Any intervention study was eligible if the wellbeing outcome was measured using the 7 or 14-item WEMWBS scale assessed both pre- and post-intervention. We identified eligible intervention studies using three approaches: a database search (Medline, EMBASE, CINAHL, PyschInfo and Web of Science from January 2007 to present), grey literature search, and by issuing a call for evidence. Narrative synthesis and random-effects meta-analysis of standardised mean differences in the intervention group were used to summarise intervention effects on WEMWBS score. Results: We identified 223 interventions across 209 studies, with a total of 53,834 participants across all studies. Five main themes of interventions were synthesised: psychological (n = 80); social (n = 54); arts, culture and environment (n = 29); physical health promotion (n = 18); and other (n = 28). Psychological interventions based on resilience, wellbeing or self-management techniques had the strongest effect on wellbeing. A broad range of other interventions were effective at improving mental wellbeing, including other psychological interventions such as cognitive behavioural therapy, psychoeducation and mindfulness. Medium to strong effects were also seen for person-centred support/advice (social), arts-based, parenting (social) and social prescribing interventions. However, a major limitation of the evidence was that only 75 (36%) of studies included a control group. Conclusions: WEMWBS has been widely used to assess wellbeing across a diverse range of interventions, settings and samples. Despite substantial heterogeneity in individual intervention design, delivery and groups targeted, results indicate that a broad range of intervention types can successfully improve wellbeing. Methodological changes, such as greater use of control groups in intervention evaluation, can help future researchers and policy makers further understand what works for mental wellbeing.

## 1. Introduction

Wellbeing has long been recognised as important for health, however only in recent years have attempts been made to conceptualise wellbeing as an outcome in health research [1] Wellbeing is a multidimensional concept of affect and psychological functioning, including both the hedonic perspective, defined as the subjective experience of happiness and life satisfaction, and the eudemonic perspective, which focuses on psychological functioning and self-realisation [2]. A widely used measure of wellbeing is the Warwick-Edinburgh Mental Wellbeing Scale (WEMWBS), which defines mental wellbeing as the positive aspect of mental health [3]. Building on previous scales, WEMWBS was developed between 2005 and 2010 within United Kingdom (UK) public mental health settings for use in Scottish population surveys and for the evaluation of projects, programmes and policies that promote mental health [4,5]. WEMWBS consists of 14 positively worded questions about an individual’s mood, interpersonal relationships and functioning over the past two weeks [2]. A shortened 7-item version was developed, focusing on the function-related questions [6].

Fifteen years since their development [3], WEMWBS scales are now used in a broad range of public health and voluntary sector settings, and have been nationally adopted to monitor mental wellbeing at the population level and develop policy [7,8]. In evaluation research, the WEMWBS scales are commonly used as an outcome in quasi-experimental and experimental designs to evaluate interventions, further strengthening the consistency and comparability of evidence for decision-making. Despite the wide use of the scales, the characteristics and quality of studies that use these measures remains relatively unknown. There is no one-size-fits-all recommendation for measuring wellbeing [9], and the substantial heterogeneity in wellbeing measurement—upwards of 100 different instruments [10]—limits our ability to understand correlates and causes of positive mental wellbeing.

Conducting a rapid systematic review and meta-analyses of all intervention studies that have measured WEMWBS as an outcome can provide insight into what works to improve wellbeing and is a first step towards understanding the methodological considerations when using the scales in different intervention and research settings. This builds on previous work that explored the use of WEMWBS scales in public health research by looking largely at the registered users of the scale between 2012 and 2016 [7]. Findings pointed to the use of evaluations of non-traditional interventions (arts-based, environmental change, community support), often in community settings, and make a case for more in-depth and systematic investigation of the potential determinants and protective factors for mental wellbeing. By focusing on WEMWBS, intervention types and themes that improve wellbeing can be identified without outcome heterogeneity, and the quality of evidence and the remaining evidence gaps can be appraised, ultimately informing future research, policy and practice.

Therefore, the aim of this project was to conduct a rapid systematic review of interventions that use WEMWBS and evaluate which interventions are the most effective at increasing mental wellbeing. Specifically, we aimed to answer the following research questions:

1. What WEMWBS-based evaluation research has been carried out to assess the effectiveness of programmes and pilots on mental wellbeing?
2. What are the key findings from the evaluation research?
3. What is the strength of evidence of the evaluation research?

## 2. Methods

This rapid review was conducted following the Preferred Reporting Items for Systematic Reviews and Meta-Analyses guidelines [11] and guidance from the Cochrane Collaboration [12]. The study protocol was registered with PROSPERO (CRD42021288606).

### 2.1. Eligibility Criteria

Studies from peer-reviewed journals and grey literature sources were eligible for inclusion if they met the following PICO (Population, Intervention, Control, and Outcome) criteria. Eligible *populations* included children and adults, with no health or age restrictions. Any *intervention* study, with or without a *control* group, was eligible if the wellbeing *outcome* was measured using the 7 or 14-item WEMWBS scales (Supplemental File S1) assessed both pre- and post-intervention. Furthermore, records must have been available in English, have sufficient detail to appraise study quality (e.g., no conference abstracts or presentation slides), and have reported on interventions taking place in the UK.

### 2.2. Search Strategy

In November 2021, we identified eligible studies using three approaches: database search, grey literature search and a Call for Evidence. Medline, EMBASE, CINAHL, PyschInfo and Web of Science were searched for all relevant articles from January 2007 to present. Our final search strategy combined different iterations of the WEMWBS acronym and scale name using truncation and wildcards as appropriate in each database: *WEMWBS OR “Warwick? Edinburgh Mental Well? being Scale”. Supplemental File S2 provides an example search strategy used in Medline. We searched the following grey literature resources: NHS Evidence, Social Science Research Network, King’s Fund Library, the Health Foundation, the Mental Health Foundation, Google Advanced Search (first 100 records). Additionally, all research papers on the Warwick Medical School WEMWBS page were screened [13]. The What Works Centre for Wellbeing (WWCW) published a Call for Evidence via their website, newsletter, social media channels and further distributed by partner members of the wellbeing research community.

### 2.3. Study Selection

Two reviewers (JMBl, JMBi or AK) independently screened 20% of all titles and abstracts; any conflicts were resolved through group discussion. A single reviewer (JMBl or JMBi) screened the remaining 80%. This approach was repeated for the second stage for full-text articles. Additionally, a second reviewer (JMBl or AK) screened all full-text articles excluded by the first reviewer to ensure no eligible study was excluded [12].

### 2.4. Critical Appraisal

The WWCW Quality Checklist: quantitative evidence of intervention effectiveness was developed by WWCW academics and the Office of National Statistics (ONS) based on the Early Intervention Foundation (EIF) Standards of Evidence [14]. The checklist assesses ten elements of study quality: fidelity, measurement, counterfactual, representativeness, sample size, attrition, equivalence, measures, analysis, and interpretation of findings (Supplemental File S3). Each element is scored as 1 (yes) or 0 (no, can’t tell, or not applicable); scores for each included record were summed to indicate low (0–2), moderate (3–6) or high (7–10) levels of confidence [15].

### 2.5. Data Extraction

A single reviewer independently extracted data, with a second reviewer checking the extracted data against the original document for 20% of papers and re-assessing any critical appraisal scores recorded as ‘can’t tell’ or ‘unsure’. The following information was extracted: record type (peer-reviewed paper or report), study sample (description, age, control group, randomisation), intervention (description, type, name), WEMWBS scale (7 or 14-item), WEMWBS scores (sample size, mean, standard deviation pre- and post-intervention score for intervention and control groups) and critical appraisal checklist. For studies that reported multiple post-intervention scores, the first score was extracted. WebPlotDigitizer was used to obtain data presented in graphs and not tables [16]. Attempts were made to contact all authors for missing information on sample size, mean and standard deviations (SD).

### 2.6. Synthesis

A narrative synthesis was conducted, following established guidelines, to describe sample characteristics, intervention types, data extraction and critical appraisal findings [17]. Intervention types were coded thematically and results were described by sub-theme. Due to heterogeneity in analytical approaches, we first synthesised the reports of positive, negative or null associations. Next, given that pre- and post-intervention scores (means ± SD) were the most commonly reported results, we conducted random-effects meta-analyses of standardised mean differences (SMD), also referred to as Hedge’s g [18], using the *meta* and *metaphor* Packages in R. Aggregate SMD effect sizes were reported for sub-themes with data from 4+ studies; 0.20, 0.50 and 0.60 correspond to small, medium and large effect sizes, respectively [19]. The *SMD* is calculated as:

$$SMD=\frac{Mean\;WEMWBS\;score_{Post-intervention}-Mean\;WEMWBS\;score_{Pre-intervention}}{Standard\;Deviation_{Pooled}}$$

For each individual meta-analysis, we measured heterogeneity using the I^2^ statistic, where >75% indicates considerable heterogeneity [18]. Where study information was unavailable, we utilised approaches recommended by the Cochrane Collaboration for dealing with missing data in meta-analyses during extraction (e.g., SD imputation, medians, ranges, interquartile ranges, etc.) [18,20].

## 3. Results

### 3.1. Search Results

**Figure 1:**
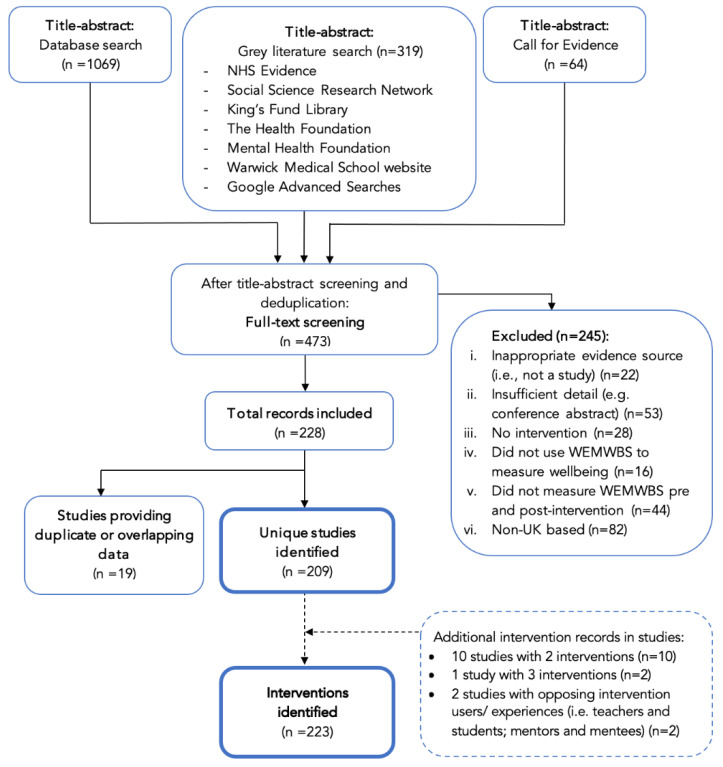
PRISMA diagram.

The search identified 1069 database records, 319 grey literature records and 64 records from the Call for Evidence. After initial title-abstract screening, 473 records underwent full-text review. Although 228 records met criteria, some reported duplicate data for the same sample and intervention, further reducing to 209 studies included in the review [21,22,23,24,25,26,27,28,29,30,31,32,33,34,35,36,37,38,39,40,41,42,43,44,45,46,47,48,49,50,51,52,53,54,55,56,57,58,59,60,61,62,63,64,65,66,67,68,69,70,71,72,73,74,75,76,77,78,79,80,81,82,83,84,85,86,87,88,89,90,91,92,93,94,95,96,97,98,99,100,101,102,103,104,105,106,107,108,109,110,111,112,113,114,115,116,117,118,119,120,121,122,123,124,125,126,127,128,129,130,131,132,133,134,135,136,137,138,139,140,141,142,143,144,145,146,147,148,149,150,151,152,153,154,155,156,157,158,159,160,161,162,163,164,165,166,167,168,169,170,171,172,173,174,175,176,177,178,179,180,181,182,183,184,185,186,187,188,189,190,191,192,193,194,195,196,197,198,199,200,201,202,203,204,205,206,207,208,209,210,211,212,213,214,215,216,217,218,219,220,221,222,223,224,225,226,227,228,229]. Supporting information from duplicate records were used to supplement data extraction and synthesis [207,230,231,232,233,234,235,236,237,238,239,240,241,242,243,244,245]. Additional data was provided by some studies for multiple interventions, giving a final 223 interventions for evidence synthesis (see Figure 1).

### 3.2. Study Characteristics

The characteristics below are described at the study level (n = 209; Table 1). Baseline sample size ranged from 4 to 4942, with a total of 53,834 participants. There were 150 peer-reviewed publications, 53 reports and 6 additional records. Most studies involved adults aged 26 to 59 (n = 175), with a third of studies examining younger adults (ages 19–25; n = 76) and a third examining older adults (aged ≥ 60; n = 63). Of 75 studies with a control group, 44 used individual or cluster randomisation to assign participants to the intervention or control conditions, 13 used a wait-list control group and 18 used neither protocol. Most studies used the 14-item WEMWBS scale (n = 145). Finally, 35 studies examined WEMWBS scores by subgroup (e.g., age, gender, ethnicity) and 66 studies assessed wellbeing at multiple follow-up points. Approximately half of the interventions were delivered to healthy community-dwelling samples and over a third to individuals with mental health difficulties, however there was substantial variability in severity, diagnosis, and description of clinical and mental health characteristics (e.g., self-diagnosed depressive symptoms vs. in-hospital patients with psychosis).

### 3.3. Key Findings by Theme

**Figure 2:**
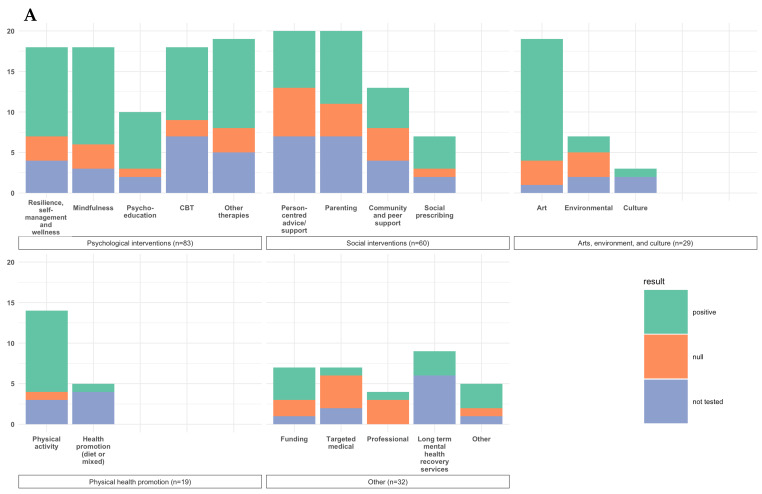

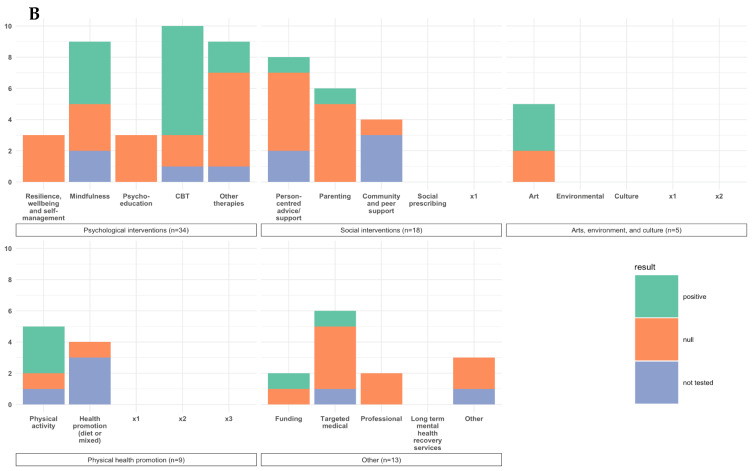
Intervention types and effect on wellbeing for: (**A**). difference in pre and post WEMWBS score in intervention group (n = 223); (**B**). difference between intervention and control group (n = 79).

Mapping of the interventions revealed four main intervention themes: (1) psychological (n = 80) [21,22,23,24,25,26,27,28,29,30,31,32,33,34,35,36,37,38,39,40,41,42,43,44,45,46,47,48,49,50,51,52,53,54,55,56,57,58,59,60,61,62,63,64,65,66,67,68,69,70,71,72,73,74,75,76,77,78,79,80,81,82,83,84,85,86,87,88,89,90,91,92,93,94,95,96,97,98,99,100]; (2) social (n = 54) [101,102,103,104,105,106,107,108,109,110,111,112,113,114,115,116,117,118,119,120,121,122,123,124,125,126,127,128,129,130,131,132,133,134,135,136,137,138,139,140,141,142,143,144,145,146,147,148,149,150,151,152,153,154,155]; (3) arts, culture and environment (n = 29) [156,157,158,159,160,161,162,163,164,165,166,167,168,169,170,171,172,173,174,175,176,177,178,179,180,181,182,183,184]; and (4) physical health promotion (n = 18) [185,186,187,188,189,190,191,192,193,194,195,196,197,198,199,200,201,202]. An additional fifth theme (‘Other’; n = 28) captured interventions that did not fall into the categories above [203,204,205,206,207,208,209,210,211,212,213,214,215,216,217,218,219,220,221,222,223,224,225,226,227,228,229]. A summary of the key findings across themes and corresponding subthemes is provided in Figure 2A; here the difference in WEMWBS score from pre to post-intervention, as reported by each study, is summarised as positive (i.e., intervention improved wellbeing), null (no association) or negative (i.e., intervention worsened wellbeing). Figure 2B provides a summary of comparisons between interventions and control groups (n = 75 studies and 79 intervention comparisons). Detailed study characteristics provided in Supplemental File S4 include: sample description, intervention details, effect of intervention on wellbeing (pre- vs. post- and compared to control, if applicable) and critical appraisal score. Next, detailed results are described for each theme.

#### 3.3.1. Theme 1: Psychological (n = 80)

Five sub-themes emerged under the psychological theme: (1) 18 interventions on resilience, self-management and wellness [21,22,23,24,25,26,27,28,29,30,31,32,33,34,35,36,37,38]; (2) 16 mindfulness intervention studies [39,40,41,42,43,44,45,46,47,48,49,50,51,52,53,54]; (3) 9 psychoeducation intervention studies [55,56,57,58,59,60,61,62,63]; (4) 18 cognitive behavioural therapy (CBT) studies [64,65,66,67,68,69,70,71,72,73,74,75,76,77,78,79,80,81]; and (5) 19 studies of other therapy interventions including Acceptance and Commitment Therapy (n = 4) [84,92,93,96], counselling (n = 4) [85,86,94,95], pet therapy (n = 2) [88,90], solution-focused brief therapy (n = 2) [97,98] and other unique therapy types [82,83,89,91,99,100]. Study details are provided in Supplemental File S4A.

Most studies with psychological interventions reported an improvement in wellbeing amongst those participating in the intervention (Figure 2). Of note, among the few studies with a control group, none of the resilience, self-management nor psychoeducation studies reported greater wellbeing improvement or post-intervention wellbeing in intervention groups compared to control [25,37,38]. In contrast, four of eight mindfulness and eight of eleven CBT interventions reported better wellbeing in the intervention groups compared to control. The largest improvements in wellbeing were in courses and programmes with a greater number of sessions (e.g., range: 4–20) taking place over a longer period (e.g., over 6+ weeks) [21,28,50,52,54,55,56,62,70].

**Figure 3:**
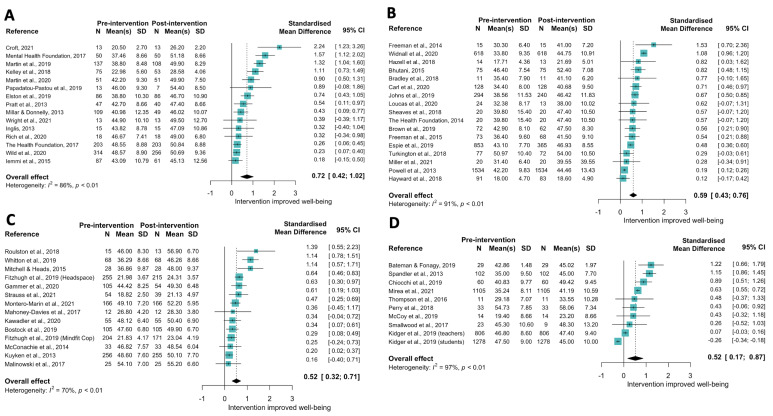
Forest plot indicating change in WEMWBS score (standardised mean difference) from pre to post intervention for the Psychological theme for (**A**). Resilience, self-management and wellness [21,22,23,24,25,26,27,28,29,31,32,33,36,37,38]; (**B**). Cognitive behavioural therapy [64,65,66,67,68,69,70,71,72,73,74,75,76,78,79,80,81]; (**C**). Mindfulness [40,41,43,44,45,46,47,48,50,51,52,53,54]; and (**D**). Psychoeducation interventions [55,56,57,58,59,60,61,62,63].

Figure 3 shows forest plots across the four main sub-themes. Fifteen of 18 resilience, self-management and wellness studies were included in the meta-analysis of standardised mean differences between pre and post-intervention, revealing a large impact of these interventions on wellbeing (SMD = 0.72 (0.42, 1.02)). Meta-analyses of 13 mindfulness interventions (SMD: 0.52 (95% CI: 0.33, 0.72)), 13 CBT interventions (SMD: 0.58 (0.42, 0.75)) and 9 psychoeducation interventions (SMD: 0.52 (0.17, 0.87)) all indicated a moderate impact on wellbeing. Of note, one study evaluating the impact of mental aid training and peer support for teachers assessed the impact on student wellbeing as a secondary outcome [57]; although student well-being scores appeared to decrease post-intervention (Figure 3D), this effect was attenuated after adjustment for baseline score, region, gender, ethnicity and free school meals. Finally, due to high heterogeneity in the other therapy types, no meta-analysis was conducted, although a forest plot of comparable data is provided in Supplemental File S5A.

#### 3.3.2. Theme 2: Social (n = 54)

Four sub-themes were identified under the social interventions theme. This included: (1) 18 studies of 20 person-centred advice/support interventions [101,102,103,104,105,106,107,108,109,110,111,112,113,114,115,116,117,118]; (2) 16 parenting interventions studies [119,120,121,122,123,124,125,126,127,128,129,130,131,132,133,134] including one study describing three different interventions [131]; (3) 12 community [144,145,146,147,148] or peer-support interventions [137,138,139,140,141,142,143]; and (4) 7 social prescribing interventions [149,150,151,152,153,154,155]. See Additional File S4B for study details.

Findings were mixed for person-centred advice/support interventions, with a third reporting a positive impact on wellbeing [101,102,106,110,116,117], a third finding no difference [103,107,108,109,115] and the remaining third did not test pre-post differences [104,105,111,112,113,114,118]. Half of the parenting programme interventions reported improved parental wellbeing post-intervention [121,122,123,125,126,127,128,132], although only one in six reported a positive impact compared to a control group [128]. There was minimal evidence to support the beneficial impact of peer support interventions (n = 1/7 [137]; n = 0/3 compared to control), with mixed evidence for community interventions which focused social or volunteering activities (n = 3/5 [144,145,146]; 0 control groups) and social prescribing interventions (n = 4/7 [151,152,153,154]; 0 control groups).

**Figure 4:**
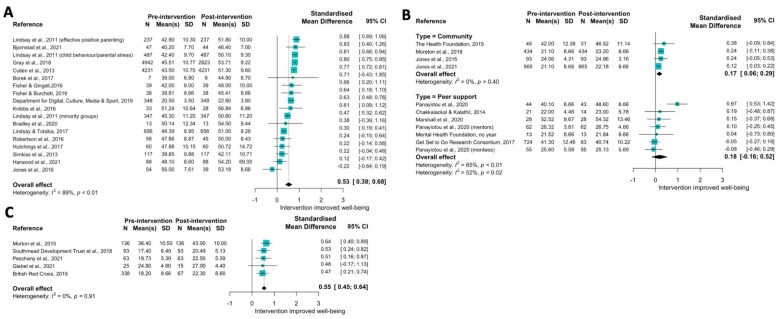
Forest plot indicating change in WEMWBS score (standardised mean difference) from pre to post intervention for the Social theme for (**A**). Parenting [119,120,121,122,123,124,125,126,127,128,129,130,131,132,133,134]; (**B**). Community and Peer support [138,139,140,141,142,143,144,145,146,148]; and (**C**). Social Prescribing interventions [151,152,153,154,155].

Figure 4 provides forest plots across three available sub-themes. Due to strong differences between interventions for person-centred advice or support interventions, an aggregate SMD was not estimated, although a parallel forest plot is available in Supplemental File S5B. All parenting programme interventions provided sufficient data for meta-analysis, which indicated a medium effect size (SMD: 0.53 (0.38, 0.68)). Community and peer-support interventions were included separately in the meta-analyses. There was no overall improvement in wellbeing in those taking part in peer support interventions (SMD: 0.18 (−0.16, 0.52)), whereas there was a small effect size for community interventions (SMD: 0.17 (0.06, 0.29)). Finally, there was a medium to high effect of social prescribing on wellbeing (SMD: 0.55 (0.45, 0.64)), with no statistical heterogeneity (0%) due to complete overlapping of confidence intervals across the five studies.

#### 3.3.3. Theme 3: Arts, Environment and Culture (n = 29)

**Figure 5:**
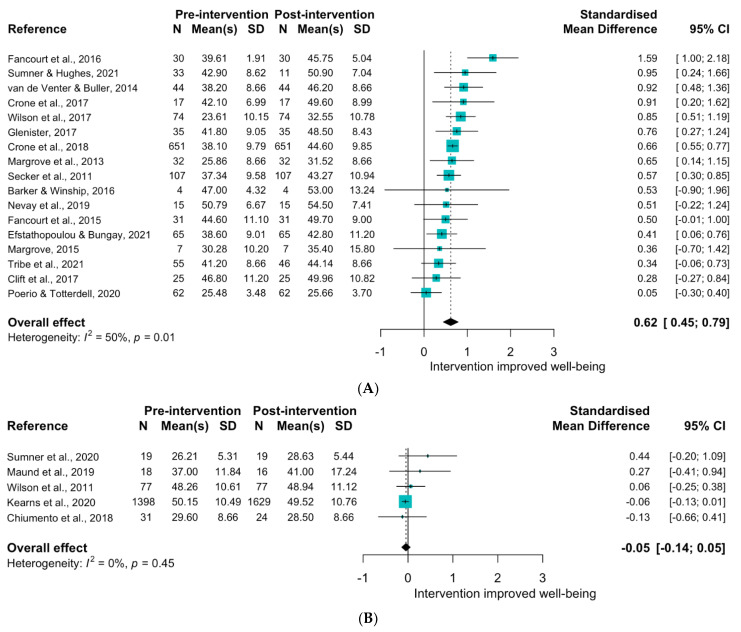
Forest plot indicating change in WEMWBS score from pre to post intervention for Theme 3: (**A**). Art [156,157,158,159,160,161,162,164,165,166,167,168,170,171,172,173,174] and (**B**). Environmental interventions [179,181,182,183,184]. Change indicated by standardised mean difference.

Nineteen studies evaluated art interventions, which included activities such as singing, music lessons, textiles, painting, drama classes, photography, fictional audiobooks and stand-up comedy (see Supplemental File S4C for study details) [156,157,158,159,160,161,162,163,164,165,166,167,168,169,170,171,172,173,174]. Art had a strong impact on wellbeing with significant improvements pre to post in more than 75% of the studies, including interventions such as stand-up comedy, listening to fictional audiobooks and two mixed visual arts classes [156,165,168]. Three of five studies reported that the intervention improved wellbeing as compared to a control group; these were all long-term interventions consisting of 10–12 weeks of weekly choir, drumming, or mixed visual art sessions [162,163,166]. The meta-analysis revealed a strong effect size (SMD: 0.62 (0.45, 0.79); Figure 5A).

None of the seven local environment improvement interventions [178,179,180,181,182,183,184] included a control group. Five studies examined if wellbeing changed over the course of the intervention, with only two reporting a significant increase [182,183]. There was no overall effect (SMD: −0.05 (−0.14, 0.05); see Figure 5B), although this was driven by null results from a large urban regeneration study (n = 1398) [181].

There were three culture-based interventions [175,176,177]. One reported that wellbeing increased in young African-Caribbean men after participation in workshops and activities in which they explored their culture and heritage [175]. The other two studies, targeting exploration of either prehistoric landscapes [176] and local arts and culture [177], did not formally assess if the interventions improved wellbeing. No study had a control group.

#### 3.3.4. Theme 4: Physical Health Promotion (n = 18)

**Figure 6:**
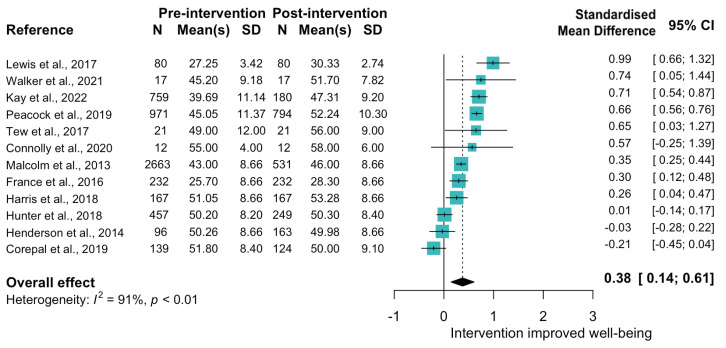
Forest plot indicating change in WEMWBS score from pre to post intervention for Theme 4: Physical activity interventions. Change indicated by standardised mean difference [185,186,187,190,191,192,193,194,195,196,197,198].

Fourteen of the eighteen physical health promotion studies were physical activity interventions [185,186,187,188,189,190,191,192,193,194,195,196,197,198]. All except for one—a football-based exercise program [191]—reported an improvement in wellbeing; conversely, the study with the largest effect size was also a football-based exercise program taking place at a professional football ground.194 Two studies assessed acute wellbeing change (tested before and after <1 h interventions) [188,189], thus were not included in further synthesis. The meta-analysis of 12 studies indicated that physical activity interventions had a moderate effect on wellbeing (Figure 6; SMD: 0.38 (0.14, 0.61)). There were mixed results when comparing interventions and control groups; two favoured improvements in the intervention group [192,197], one found no effect [185], and one did not test differences [186].

Of the remaining four health promotion interventions, two focused on alcohol screening and education in adolescents [199,200], one on exercise and diet workshops [201], and one was multi-disciplinary, supporting participants to achieve a healthy lifestyle with a focus on alcohol use, smoking, diet and physical activity [202]. The exercise-diet workshop had a positive impact on wellbeing [201], whereas alcohol education did not improve wellbeing, compared to those who did not receive the information [200]. The other two studies did not test differences over time nor between control and intervention groups. An additional four national-level health promotion interventions are described under the funding section below [203,204,205,206]. See Additional File S4D for study details.

#### 3.3.5. Theme 5 Other (n = 28)

Interventions that did not clearly fit into one of the four main themes are described below. This includes: funding (n = 7) [203,204,205,206,207,208,209], targeted medical interventions (n = 7), recovery colleges (n = 5), professional training (n = 4), and other (n = 5). See Supplemental File S4E for study details. Large scale funding programmes included: a lifestyles and community wellbeing programme (positive effect [203,204]), older adults’ physical activity and diet (positive effect [205,206]), youth services (positive [207]; null [208]), and troubled families [209]. Consistent with other sub-themes, no meta-analysis was conducted due to substantial differences in programmes; individual study estimates are shown in Supplemental File S5C. Seven interventions had a medical aim (e.g., targeting vision, hearing, memory, physical function, or cardiovascular disease), with just two reporting subsequent improvements in wellbeing. This included Celecoxib augmentation (typically used to treat pain) in those with an anxiety disorder [212] and faster access to a hearing dog for those with hearing loss [216]. Five studies investigated if attending recovery colleges or personalised mental health residential services improved wellbeing [220,221,222,223,224]. Although none had a control group, three services had a positive impact on wellbeing [220,221,224]. Four studies evaluated professional interventions that delivered training to healthcare practitioners [217,218], healthcare managers and employees [219], and frontline domestic abuse practitioners [135]. Just one intervention, a 2-day course teaching health practitioners how to help patients with mental health or learning difficulties develop social networks, had a positive impact on wellbeing [218]. Other interventions that did not fit into previous themes included: couples massage classes (positive) [225], sleep education programme for parents (positive) [226], co-design of workplace solutions (positive) [227], social media restriction for university students (null) [228] and small-scale aids/home adaptations for dementia patients (null) [229].

### 3.4. Critical Appraisal

A total of 46.4% of studies scored as high quality (n = 97; 7–10 points), 53.1% as moderate (n = 111; 3–6 points), and one as low quality (0.5%; 0–2 points). The checklist (Supplemental File S2) is likely to have overestimated study quality due to the review eligibility criteria and binary scoring of each element, therefore individual items are explored below.

Fidelity was high amongst 193 (92%) of the studies, with only 16 studies failing to clearly describe intervention details. Second, the minimum sample size required 20 participants to have completed the measures pre- and post-intervention; this was met by most studies (n = 159; 76%). Third, 205 studies (98%) received 1 point for the measures criteria—using a standardised, validated measure published independently of the study—as they used an unmodified WEMWBS scale. The four modifications to the scale included reworded ‘wellbeing check cards’ for 9–15 year olds [179], simplified language for those with learning disabilities [46], a printing error that omitted one item [62] and grouping of individual WEMWBS items with other questions [180]. Note that these four evaluations were excluded from both the narrative synthesis and meta-analyses due to improper use of the scale. Next, the most common analytical approach was consistent with that recommended on the Warwick Medical School website [246]: calculating and comparing means and standard deviations using a t-test. Eighty-four percent (n = 176) either examined statistical differences in means or presented other appropriate statistical results (e.g., regressions). Finally, a positive score on the consistency criterion (n = 197; 94.3%) reflected explicit findings and consistency between results and discussion.

The other five checklist elements had a lower distribution of scores. As the majority of studies did not have control groups, scores on counterfactual (n = 57; 27%) and equivalence (n = 52; 25%) were low. The lack of control groups shifts the summary of evidence substantially (Figure 2B). Fewer than half of studies (n = 101; 48%) received a point for being representative of the target population. Although studies with control groups commonly demonstrated similar characteristics between the control and intervention groups, studies without a control group often failed to assess if the sample was representative relative to the target population. Another key area of concern was measurement (n = 98; 47%) as many studies examined those who completed the intervention, ignoring any lost to follow-up. The final element of the critical appraisal checklist was attrition (n = 108, 52%). Despite a low attrition criterion (≥35% completing pre and post-measures), many studies failed to report drop-out and did not compare characteristics between those who completed the intervention and those who dropped out.

## 4. Discussion

### 4.1. Key Findings

In this comprehensive rapid systematic review, we identified 223 interventions across 209 studies that used WEMWBS to assess improvements in wellbeing. Five themes of intervention were identified: psychological; social; arts, environment and culture; health promotion; and other. Synthesis across all themes revealed that a broad range of interventions can positively improve wellbeing, however interventions based on resilience, self-management and wellness techniques had the greatest impact on wellbeing. Other interventions with medium to large effects included those related to art, support/advice (e.g., person-centred, parenting) or psychological aspects (e.g., CBT, social prescribing, psychoeducation, mindfulness). Physical activity and community-based interventions had a small effect. There was no evidence that peer-support or environmental interventions altered wellbeing. See Table 2 for summary of SMDs by intervention type. Although the WWCW Quality Checklist indicated moderate-high quality of evidence across studies, the critical appraisal section highlighted the main limitations including the inclusion of control groups in only 35% of all intervention evaluations, which altered the summary of evidence (Supplemental File S6). For example, although interventions on resilience, self-management and wellness had the largest SMD (see Table 2), no study found that wellbeing improved in the intervention in comparison to the control group (n = 3 null, n = 0 positive).

### 4.2. Comparison to Other Reviews

Other reviews of wellbeing interventions have also highlighted heterogeneity of intervention type, sample and setting as major limitations [1,248,249,250,251]. Additionally, these reviews report substantial heterogeneity in wellbeing outcome measures, which limits synthesis and meta-analyses of results [1,248,249,250]. Our findings are largely consistent with other reviews including those that have focused primarily on psychological interventions in isolation [1,250] and those who compared to other themes [251,252,253,254]. For example, a recent review of 419 psychological intervention RCTs, with 48 different wellbeing outcome measures, reported the largest effect sizes for mindfulness and positive psychological (comparable to resilience/self-management/wellness), followed by CBT and other therapies [1]. A review of workplace-based interventions also found that psychological interventions, one of six identified themes, had the greatest improvement on wellbeing [251]. Several reviews have also demonstrated the efficacy of such interventions in specific settings such as work or school [251,252,253,254]. By eliminating heterogeneity in wellbeing measurement, our review allowed effect sizes to be compared across intervention type.

### 4.3. Sources and Explanations of Heterogeneity

Clinical and methodological heterogeneity across studies resulted from differences in sample characteristics, baseline WEMWBS scores, frequency and duration of interventions and primary aims of interventions. Although the SMD provides an overall indication of intervention success, identifying the components of a successful intervention (e.g., demographic, setting, length, frequency and duration) was not possible. Yet, differences in intervention design and delivery may explain conflicting finding within single themes. For example, engagement intensity of peer-centred advice interventions ranged from single incident peer-led advice on welfare benefits and health advocacy [116], to intensive ongoing advice services over a 2-month to 2-year period [106]. Despite the use of a random-effects meta-analysis to partially account for study differences, the majority of I2 statistics indicated high statistical heterogeneity. Due to the breadth of studies captured in this review, it is unsurprising that certain interventions had substantially larger impacts than others on wellbeing. Bigger improvements in wellbeing were commonly observed in studies with longer interventions (e.g., weekly sessions for 6–12 weeks) compared to single sessions [50,52,54,55,56,62,70,162,163,166].

### 4.4. Implications for Research and Policy

The WEMWBS scales have been used to evaluate a wide range of interventions, offering a common unit of benefit to compare effectiveness across social policy and service areas. The positive impact of most intervention types suggests that a wide range of interventions to improve wellbeing should be supported. However, more research on intervention topic (e.g., such as learning resilience techniques or improving sleep) and modes of delivery (e.g., frequency, duration, group-based or one to one) would provide a better understanding of the key ingredients of intervention success to inform decision-making. In addition, where studies examine effectiveness by subgroups, an analysis of potentially different impacts of interventions on specific population groups would help inform equity considerations in policy and funding. Finally, information on intervention efficiency would allow for cost effectiveness comparisons to inform investments and spending decisions.

Researchers and evaluators should implement higher quality designs where possible, using control groups and attempting intention-to-treat analysis to improve the strength of findings. Waitlist control groups may represent a feasible option where controlled designs are more challenging (e.g., recovery colleges) and investigating the representativeness of samples compared to target populations would greatly improve the confidence in findings.

### 4.5. Strengths and Limitations

This comprehensive rapid systematic review followed a rigorous registered protocol with a simple and inclusive search strategy which maximised identification of relevant records. Grey literature sources were searched, and a successful Call for Evidence increased the pool of evidence and minimised publication bias. Missing data was reduced by contacting authors. Finally, we followed recent guidance from the Cochrane collaboration to conduct the rapid review process [12]; double screening of all full-text articles excluded by the first reviewer ensured that no studies were inadvertently omitted from the review.

There are several limitations that must be acknowledged in relation to the rapid review and meta-analysis methodology. First, only English-language UK-based studies were included, and records with insufficient study detail (e.g., conference abstracts, and presentation slides) were excluded. Second, a single reviewer screened and extracted most of the data, although quality assurance processes were in place to reduce errors. Next, the meta-analysis was limited to SMD with no meta-analysis of mean change difference between control and intervention group, nor a meta-regression of studies providing model estimates, however there were insufficient studies across themes to assess either of these analyses. Furthermore, we did not investigate how associations between interventions and wellbeing differed by participant characteristics (age, gender, ethnicity, mental health status, etc.) or changed across different lengths of follow-up.

## 5. Conclusions

This rapid review summarises the key findings of mental wellbeing interventions with WEMWBS measurements conducted over the past 15 years. Revisiting our key research questions, we first identified 223 interventions (209 studies) that have assessed the effectiveness of various programmes or pilots on mental wellbeing. Next, synthesis of this evaluation research provided strong evidence that a broad range of interventions are effective at improving mental wellbeing, with medium to strong effects shown for psychological, social and art interventions. Finally, the quality of the evidence and the heterogeneity between individual intervention design, delivery and target group made it challenging to draw strong conclusions, particularly in the absence of a control group in many studies. Policy makers would benefit from more robust studies to increase the availability of higher-quality comparable evidence on what works to improve mental wellbeing. Further research should prioritise thematic areas where evidence appears more scarce, or of a lower quality, as well as investigating cost-effectiveness and equitable impact of wellbeing interventions.

## Figures and Tables

**Table 1:** Characteristics of all included studies (n = 209).

	N (%)
**Evidence type**	
Peer-reviewed publication	150 (71.8)
Report	53 (25.4)
Other (e.g., evaluation summaries and evidence briefings)	6 (2.0)
**Age group ^a^**	
Children (0–10)	3 (1.4)
Adolescents (11–18)	22 (10.5)
Young adults (19–25)	76 (36.4)
Adults (26–59)	175 (83.7)
Older adults (60+)	63 (30.1)
**Control group**	
No	132 (63.2)
Yes	77 (36.8)
**Randomisation (for studies with control group)**	
Individual randomisation	33 (44.0)
No randomisation nor wait-list	18 (24.0)
Wait-list control group	13 (17.3)
Cluster randomisation	11 (14.7)
**Wellbeing measure**	
14-item WEMWBS	145 (69.4)
7-item SWEMWBS	64 (30.6)
**Examined WEMWBS scores by subgroup**	
No	174 (83.3)
Yes	35 (16.7)
**Assessment at additional follow-up points**	
No	143 (68.4)
Yes	66 (31.6)

^a^ Percentages do not add up to 100% due to multiple age groups in studies (48% of studies).

**Table 2:** Ranked summary of overall standardised mean difference (SMD) by intervention type.

Theme	Intervention Subtheme	SMD (95% Confidence Intervals) ^a^
Psychological	Resilience, self-management and wellness	0.72 (0.42, 1.02)
Arts, Environment & Culture	Art	0.62 (0.45, 0.79)
Social	Person-centred support and advice	0.58 (0.14, 1.02)
Psychological	CBT	0.58 (0.42, 0.75)
Social	Social prescribing	0.55 (0.45, 0.64)
Social	Parenting	0.53 (0.38, 0.68)
Psychological	Psychoeducation	0.52 (0.17, 0.87)
Psychological	Mindfulness	0.51 (0.33, 0.72)
Physical health promotion	Physical activity	0.38 (0.14, 0.61)
Social	Peer-support	0.18 (−0.16, 0.52)
	Community-based	0.17 (0.06, 0.29)
Arts, Environment & Culture	Environment	−0.05 (−0.14, 0.05)

^a^ >0.60 indicates a large effect size, >0.50 indicates a medium effect size, and >0.20 indicates a small effect size [19,247].

## Data Availability

Not applicable.

## Supplementary Materials

The following supporting information can be downloaded at: https://www.mdpi.com/article/10.3390/ijerph192315845/s1.
