# Supplementary material for: What Works to Improve Wellbeing? A Rapid Systematic Review of 223 Interventions Evaluated with the Warwick-Edinburgh Mental Well-Being Scales
Source: Int J Environ Res Public Health. 2022 Nov 28;19(23):15845. doi: 10.3390/ijerph192315845 (PMC9737992; doi:10.3390/ijerph192315845)
Supplement: Supplementary file 1 [file ijerph-19-15845-s001.zip › ijerph-2005086-supplementary.pdf]

Supplemental File S1: The Warwick-Edinburgh Mental Wellbeing Scales from Tennant et al.[2]

The Warwick-Edinburgh Mental Wellbeing Scale (14-items): Below are some statements about feelings and thoughts. Please tick the box that best describes your experience of each over the last 2 weeks

	None of the time (1)	Rarely (2)	Some of the time (3)	Often (4)	All of the time (5)
1. I’ve been feeling optimistic about the future	○	○	○	○	○
2. I’ve been feeling useful	○	○	○	○	○
3. I’ve been feeling relaxed	○	○	○	○	○
4. I’ve been feeling interested in other people	○	○	○	○	○
5. I’ve had energy to spare	○	○	○	○	○
6. I’ve been dealing with problems well	○	○	○	○	○
7. I’ve been thinking clearly	○	○	○	○	○
8. I’ve been feeling good about myself	○	○	○	○	○
9. I’ve been feeling close to other people	○	○	○	○	○
10. I’ve been feeling confident	○	○	○	○	○
11. I’ve been able to make up my own mind about things	○	○	○	○	○
12. I’ve been feeling loved	○	○	○	○	○
13. I’ve been interested in new things	○	○	○	○	○
14. I’ve been feeling cheerful	○	○	○	○	○

The Short Warwick-Edinburgh Mental Wellbeing Scale (7-items): Below are some statements about feelings and thoughts. Please tick the box that best describes your experience of each over the last 2 weeks

	None of the time (1)	Rarely (2)	Some of the time (3)	Often (4)	All of the time (5)
1. I’ve been feeling optimistic about the future	○	○	○	○	○
2. I’ve been feeling useful	○	○	○	○	○
3. I’ve been feeling relaxed	○	○	○	○	○
4. I’ve been dealing with problems well	○	○	○	○	○
5. I’ve been thinking clearly	○	○	○	○	○
6. I’ve been feeling close to other people	○	○	○	○	○
7. I’ve been able to make up my own mind about things	○	○	○	○	○

Supplemental File S2: Example search strategy used in Medline

1	**Warwick Edinburgh Mental Wellbeing Scale.mp.** [mp=title, abstract, original title, name of substance word, subject heading word, floating sub-heading word, keyword heading word, organism supplementary concept word, protocol supplementary concept word, rare disease supplementary concept word, unique identifier, synonyms]
2	**Warwick-Edinburgh Mental Wellbeing Scale.mp.** [mp=title, abstract, original title, name of substance word, subject heading word, floating sub-heading word, keyword heading word, organism supplementary concept word, protocol supplementary concept word, rare disease supplementary concept word, unique identifier, synonyms]
3	**Warwick-Edinburgh Mental Well-being Scale.mp.** [mp=title, abstract, original title, name of substance word, subject heading word, floating sub-heading word, keyword heading word, organism supplementary concept word, protocol supplementary concept word, rare disease supplementary concept word, unique identifier, synonyms]
4	**Warwick Edinburgh Mental Well-being Scale.mp.** [mp=title, abstract, original title, name of substance word, subject heading word, floating sub-heading word, keyword heading word, organism supplementary concept word, protocol supplementary concept word, rare disease supplementary concept word, unique identifier, synonyms]
5	***WEMWBS.mp**. [mp=title, abstract, original title, name of substance word, subject heading word, floating sub-heading word, keyword heading word, organism supplementary concept word, protocol supplementary concept word, rare disease supplementary concept word, unique identifier, synonyms]
6	***SWEMWBS.mp**. [mp=title, abstract, original title, name of substance word, subject heading word, floating sub-heading word, keyword heading word, organism supplementary concept word, protocol supplementary concept word, rare disease supplementary concept word, unique identifier, synonyms]
7	1 OR 2 OR 3 OR 4 OR 5

Note: depending on capability of the database search, wildcards (?) were used to replace the "-" in Wellbeing and Warwick Edinburgh. The use of '*' allows for WEMWBS or SWEMWBS to be captured, and the wildcard '?' will address any differences in spacing or hyphenation used by authors.

Supplemental File S3: What Works Centre for Wellbeing Quality Checklist: Quantitative evidence of intervention effectiveness

The checklist below is from the quality checklist for quantitative evidence of intervention effectiveness. In a previous review, WWCW developed a scoring system has been added to provide an indication of overall level of confidence in the design, conduct and reporting of the study. The 10 elements of the checklist can be scored either 1 (yes) or 0 (no, can't tell or N/A). The total score can be used to assign each study an overall level of confidence of low (0-2), moderate (3-6) or high (7-10).

Question	Element	Response options
Was the evidence well-designed?	Fidelity:<br>• The extent to which the intervention was delivered with fidelity is clear – i.e., if there is a specific intervention which is being evaluated, this has been well reproduced.	Yes (1)<br>No (0)<br>Can't tell (0)<br>N/A (0)
	Measurement:<br>• The measures are appropriate for the intervention's anticipated outcomes and population.<br>• Participants completed the same set of measures once shortly before participating in the intervention and once again immediately afterwards.<br>• An 'intent-to-treat' design was used, meaning that all participants recruited to the intervention participated in the pre/post measurement, regardless of whether or how much of the intervention they received, even if they dropped out of the intervention (this does not include dropping out of the study - which may then be regarded as missing data).	Yes (1)<br>No (0)<br>Can't tell (0)<br>N/A (0)
	Counterfactual:<br>• Assignment to the treatment and comparison group was at the appropriate level (e.g., individual, family, school, community).<br>• The comparison condition provides an appropriate counterfactual to the treatment group. Consider:<br>• Participants were randomly assigned to the treatment and control group through the use of methods appropriate for the circumstances and target population OR sufficiently rigorous quasi-experimental methods (regression discontinuity, propensity score matching) were used to generate an appropriately comparable sample through non-random methods.<br>• The treatment and comparison conditions are thoroughly described.	Yes (1)<br>No (0)<br>Can't tell (0)<br>N/A (0)
Was the study carried out appropriately? Including appropriate sample	Representative:<br>• The sample is representative of the intervention's target population in terms of age, demographics and level of need. The sample characteristics are clearly stated.<br>• There is baseline equivalence between the treatment and comparison group participants on key demographic variables of interest to the study and baseline measures of outcomes (when feasible).	Yes (1)<br>No (0)<br>Can't tell (0)<br>N/A (0)
	Sample size:<br>• The sample size is sufficiently large to test for the desired impact. This depends most importantly on the effect size, however a suggestion could be, for example, that a minimum of 20 participants have completed the measures at both time points within each study group.	Yes (1)<br>No (0)<br>Can't tell (0)<br>N/A (0)
	**Attrition:**	Yes (1)<br>No (0)

Question	Element	Response options
	• A minimum of 35% of the participants completed pre/post measures. Overall study attrition is not higher than 65%.<br>• The study had clear processes for determining and reporting drop-out and dose. Differences between study drop-outs and completers were reported if attrition was greater than 10%.<br>• The study assessed and reported on overall and differential attrition.	Can't tell (0)<br>N/A (0)
	**Equivalence:**<br>• Risks for contamination of the comparison group and other confounding factors have been taken into account and controlled for in the analysis if possible.<br>• Participants were blind as to their assignment to the treatment and comparison group.<br>• There was consistent and equivalent measurement of the treatment and control groups at all points when measurement took place.	Yes (1)<br>No (0)<br>Can't tell (0)<br>N/A (0)
	**Measures:**<br>• The measures used were valid and reliable. This means that the measure was standardised and validated independently of the study, and that the methods for standardisation were published. Administrative data and observational measures may also have been used to measure programme impact, but sufficient<br>• Information was given to determine their validity for doing this.<br>• Measurement was independent of any measures used as part of the treatment.<br>• In addition to any self-reported data (collected through the use of validated instruments), the study also included assessment information independent of the study participants (e.g., an independent observer, administrative data etc)	Yes (1)<br>No (0)<br>Can't tell (0)<br>N/A (0)
Was the analysis appropriate?	• The methods used to analyse results are appropriate given the data being analysed (categorical, ordinal/ratio, parametric/non-parametric, etc.) and the purpose of the analysis.<br>• Appropriate methods have been used and reported for the treatment of missing data.	Yes (1)<br>No (0)<br>Can't tell (0)<br>N/A (0)
Is the evidence consistent?	• Are the findings made explicit?<br>• Is there adequate discussion of the evidence both for and against the researcher's arguments?<br>• Has the researcher discussed the credibility of their findings (e.g., triangulation, respondent validation, more than one analyst)?<br>• Are the findings discussed in relation to the original research question?	Yes (1)<br>No (0)<br>Can't tell (0)<br>N/A (0)

Supplemental File S4A: Description of studies: Psychological interventions

Reference	Participants[a]	Intervention details	Effect of intervention on wellbeing[a]		Level of confidence
			Pre- vs. Post	Compared to control	
**Resilience, wellbeing, and self-management**					
Croft, 2021[21]	Staff working in a large Social Services team (n=13)	Reconnect to Innate Resilience: 6x 90min online didactic sessions to improve and educate about resilience over an 8 week period	Significant (wellbeing increased)		Moderate
Elston et al., 2019[22]	Older people with complex multimorbidity (n=86)	Participants receive an initial 30-40 minute session with a co-ordinator (non-healthcare staff with training in goalsetting) to set goals for 'living well'. Co-ordinator then works with participant for next 12-weeks in resilience-focused coaching and advocating for local support services	Significant (wellbeing increased)		High
Iemmi et al., 2015[23]	Adults with severe mental disorders who use secondary mental health services. (n=87)	Self-management Intervention.: A 2-day peer-led self-management workshop to teach goal-setting and problem-solving techniques, to empower people and to facilitate meeting with others and sharing of experiences. The workshop was followed by 6 half day workshops over three months, and 6 on-going peer group meetings over 6 months.	Significant (wellbeing increased)		Moderate
Inglis, 2013[24]	Individuals seeing a mental health practitioner in Kirkintilloch (Scotland) (n=15)	Personal Asset Mapping: 1 to 1 session with a mental health practitioner developing a personal asset map (resources individuals have at their disposal) in a 1:1 setting	Not tested		Moderate
Kelley et al., 2018[25]	Prisoners resident in HM Prison Onley (n=114)	Three Principles Correctional Counselling (3PCC): 10 weekly 3-hour classes on three Principles Correctional Counselling aimed to sustain inner health (3 principles: Universal Mind, consciousness, and thought)	Significant (wellbeing increased)	Not significant	Moderate
Martin et al., 2019[26]	Parents of children with developmental disorders (n=137)	HOPE: 6 weekly 2.5hr self-management sessions	Significant (wellbeing increased)		Moderate
Martin et al., 2020[27]	People living with cancer or recent cancer survivors (n=51)	iHOPE (Help to Overcome Problems Effectively): 6-week web-based course consisting of text, images, downloadable documents, and links to external websites, interactive activities (e.g., quizzes, self-monitoring tools, and diaries), forums and messaging facilities	Significant (wellbeing increased)		Moderate
Mental Health Foundation, 2017[28]	Vulnerable adult male prisoners at HM Prison & Young Offers' Institution Parc in Wales (n=50)	Weekly 2-3hr session for 4 weeks that was peer-facilitated and focus on: positive thinking, goal setting, managing wellbeing and behaviour, working with professionals, developing a personal 'toolbox'	Significant (wellbeing increased)		Moderate
Millar & Donnelly, 2013[29]	Adults showing signs of mental health difficulties (n=109)	12 weekly 2hr sessions aiming to raise awareness and knowledge about how to protect mental health and wellbeing and to increase skills and personal resources	Significant (wellbeing increased)		Moderate
Mind Cymru, 2018[30]	Older adults in urban and rural areas of Wales (n not reported)	My Generation: 8 weekly 1hr sessions of a resilience training programme	Not tested		Moderate
Papadatou-Pastou et al., 2019[31]	Postgraduate and undergraduate students with minor to moderate psychological difficulties (n=13)	MePlusMe: A single full day workshop where university students were introduced to the MePlusMe system, an online psychological wellbeing and study skills support system and students were encouraged to revisit meaning material and practice what they had learnt over the follow up period	Not tested		Moderate
Pratt et al., 2013[32]	People with lived experience of mental health difficulties (n=47)	Wellness Recovery Action Planning (WRAP): Weekly sessions (split over 2 or 4 weeks) of Wellness Recovery Action Planning, which is a tool for self-management and wellness planning	Not tested		Moderate

Reference	Participants[a]	Intervention details	Effect of intervention on wellbeing[a]		Level of confidence
			Pre- vs. Post	Compared to control	
Rich et al., 2020[33]	Students training to be medical doctors (n=18)	Individualised Wellbeing And Resilience for DoctorS (iWARDS): Brief in-person workshops to enhance self-care skills and help them manage their work-life balance and use of technology	Not significant		High
Robinson et al., 2019[34]	People with long-term health conditions (diabetes, heart disease, and arthritis) (n=160)	6 weekly 2hr sessions of a mental health resilience course delivered by the charity Mind	Significant (wellbeing increased)		High
Robinson et al., 2015[35]	Unemployed men (n=53)	Local Resilience (by Mind): 5 distinct projects delivered by local Mind centres aiming to improve resilience; projects lasted 2 weeks to 6 months consisting of various physical activity, arts, CBT, psychoeducation, and mindfulness elements	Significant (wellbeing increased)		High
The Health Foundation, 2017[36]	Patients living with long-term health conditions, their families and NHS staff (n=203)	Optimising Strength & Resilience: One day workshop focusing on education, awareness-raising and behavioural change activities; committee work, change champions and strategy development were also part of the programme.	Significant (wellbeing increased)		High
Wild et al., 2020[37]	Police, ambulance, fire, and search and rescue services personnel (n=427)	Mind Resilience Program: 6-week course consisting of a weekly 2.5hr group session providing information about mental health and experiential exercises drawn from stress management and mindfulness	Not significant	Not significant	High
Wright et al., 2021[38]	People living with cancer or recent cancer survivors (n=26)	iHOPE (Help to Overcome Problems Effectively): 6-week web-based course consisting of text, images, downloadable documents, and links to external websites, interactive activities (e.g., quizzes, self-monitoring tools, and diaries), forums and messaging facilities	Not significant	Not significant	High
**Mindfulness**					
Beshai et al., 2016[39]	Secondary school teachers and staff (n=89)	.b Foundations Course: 9 sessions (one presentation and 8 weekly 75-minute sessions) on mindfulness delivered by trained teachers	Significant (wellbeing increased)	Not significant	Moderate
Bostock et al., 2019[40]	Healthy employees from two large UK companies (n=186)	Mindfulness app that offered 45 pre-recorded 10-20 minute guided audio meditations, participants asked to complete one meditation per day over 8-weeks	Significant (wellbeing increased)	Significant (wellbeing increased)	High
Fitzhugh et al., 2019[41]	All police employees across five participating forces (Avon and Somerset, Bedfordshire, Cambridgeshire, Hertfordshire, and South Wales) (n=605)	Headspace: Commercially available mindfulness app. User can decide which type and length of session to complete at any time, with no set route for completion of sessions.	Significant (wellbeing increased)	Not tested	High
	as above (n=204)	Mindfit Cop: A bespoke online 8-week self-paced mindfulness course for policing which offers mindfulness-videos, audios and documents using policing examples	Significant (wellbeing increased)	Not tested	as above
Flynn et al., 2020[42]	Carers of people with intellectual disabilities	Be Mindful: Online mindfulness program which involves 10 online pre-recorded audio and video sessions	Significant (wellbeing increased)		High
	as above (n not reported)	Be Mindful+: In addition to main online mindfulness program, the second intervention group also had a trained peer mentor for support (3 x30min telephone calls)	Significant (wellbeing increased)		as above
Gammer et al., 2020[43]	Mothers of infants under 1-year old (n=206)	Kindness for Mums Online: Interactive, online compassion-based intervention for new mothers, delivered over 5-to-6 weeks with one weekly 10-15min session	Not tested	Significant (wellbeing increased)	High

Reference	Participants[a]	Intervention details	Effect of intervention on wellbeing[a]		Level of confidence
			Pre- vs. Post	Compared to control	
Kawadler et al., 2020[44]	Healthy adults recruited via a recruitment agency who screened positive for stress (n=55)	The BioBase Programme: Participants were monitored for 4 weeks while wearing a wrist-worn BioBeam activity and heart rate monitor and to use the BioBase digital therapy app (e.g. deep breathing exercises, mood tracking, data from monitor) for at least 5 minutes per day and complete at least 14 daily 3-5minute modules sessions.	Significant (wellbeing increased)		Moderate
Kuyken et al., 2013[45]	Young people aged 12-16 in 12 secondary schools (n=522)	The Mindfulness In Schools Programme: 9 weekly scripted lessons on mindfulness skills taught by teachers trained in the MiSP	Not tested	Significant (wellbeing increased)	Moderate
Mahoney-Davies et al., 2017[46]	Adults with learning disabilities who attend a council-funded day support service (n=12)	10 weekly 2hr sessions to teach the Five Ways to Well-being (being with people, being active, noticing things around you, keep learning, giving to others)	Not significant		Moderate
Malinowski et al., 2017[47]	Older adults (n=50)	Minimum of 10 min/day of meditation at least 5 times/week for 8 weeks and 4 90-min group sessions of psychoeducation, inquiry-based discussion and group mediation practice	Not significant	Not significant	High
McConachie et al., 2014[48]	Support staff from care organisations working with individuals with intellectual disabilities who displayed challenging behaviour (n=65)	One day acceptance and mindfulness workshop	Not tested	Not significant	High
Millar et al., 2020[49]	In-patients within the psychiatric rehabilitation service (n not reported)	20-30min mindfulness group sessions 3x a week for 5 months or weekly over 18 months, followed by reflection and discussion	Not significant		Moderate
Mitchell & Heads, 2015[50]	Adults with a range of chronic psychological issues (e.g. depression, anxiety, pain, PTSD, personality disorder) (n=28)	Living Mindfully Mindfulness-Based Stress Reduction Programme: 5 weekly 2.5hr sessions providing training in 4 mindfulness practices	Significant (wellbeing increased)		Moderate
Montero-Marin et al., 2021[51]	Secondary school teachers (n=166)	8 sessions of mindfulness training with 20 minutes of a daily home mindfulness practice	Significant (wellbeing increased)		High
Roulston et al., 2018[52]	Undergraduate social work students (n=30)	Six weekly Mindfulness sessions delivered by an experienced Mindfulness trainer	Significant (wellbeing increased)	Significant (wellbeing increased)	Moderate
Strauss et al., 2021[53]	People with moderate-to-severe depression (n=54)	Clinician supported use (6 weekly sessions) of the mindfulness smartphone app, Headspace, where 30 sessions could be completed within the 60-day follow-up period	Significant (wellbeing increased)		Moderate
Whitton et al., 2019[54]	Patients at a mental health day hospital in Fife, Scotland (n=68)	8-weekly 2-hour mindfulness course delivered by clinician researchers	Significant (wellbeing increased)		High
**Psychoeducation**					
Bateman & Fonagy, 2019[55]	Families of people with Borderline Personality Disorder (n=56)	Mentalization-Based Treatment Families and Carers Training Support (MBT-FACTS): Five 1-1.5-hour sessions delivered by family members who have been trained to deliver psychoeducation, mentalization and mindfulness-based exercises, and problem-solving skills	Significant (wellbeing increased)	Not significant	High
Chiocchi et al., 2019[56]	Carers of people with severe and enduring mental illness (n=60)	Up to 20 2-hour psychosocial education sessions for carers	Significant (wellbeing increased)		High
Kidger et al., 2021[57]	Teachers and year 8 students in 25 mainstream secondary schools in South West England and Central South/South East Wales (n=1722)	Well-being in Secondary Education (WISE): Consisted of: i) 2-day standard Mental Health First Aid (MHFA) course for 8% of staff; ii) 1-day MHFA course for teachers in pastoral roles; iii) 1- hour mental health awareness session offered to all staff [WEMWBS assessed in teachers]	Not tested	Not significant	High

Reference	Participants[a]	Intervention details	Effect of intervention on wellbeing[a]		Level of confidence
			Pre- vs. Post	Compared to control	
	as above (n=2700)	as above [WEMWBS assessed in students]	Not tested	Not significant	as above
McCoy et al.,2019[58]	Parents and carers who either had an adverse childhood experience or their child did (n=14)	Adverse Childhood Experiences (ACEs) Recovery Toolkit Programme: Trained facilitators, who had attended a two-day training workshop, delivered the Adverse Childhood Experiences (ACEs) Toolkit to parents/carers who had experienced ACEs to further their understanding of how ACEs could impact them and their children and used a trauma-based psychoeducation approach so parents could develop their resilience and strategies to reduce the potential impact of ACEs on children	Significant (wellbeing increased)		Moderate
Mirea et al., 2021[59]	Adults presenting with depressive symptoms (n=1105)	Participants completed a comprehensive 635 question web-based mental health assessment over 6 sessions. They then received a results report suggesting likely mood and comorbid disorders as well as tailored psychoeducation about relevant disorders and help sources.	Significant (wellbeing increased)		High
Perry et al., 2018[60]	Carers in the Charedi Orthodox Jewish community that came in to contact with mental health in their capacity as a caregiver (n=33)	4 sessions of a culturally tailored psychoeducational group intervention delivered by a Rabbi and psychotherapist focusing on mental health promotion and prevention.	Significant (wellbeing increased)		Moderate
Smallwood et al., 2017[61]	Caregivers of patients in psychosis services (n=23)	Caregivers of people with psychosis were offered i) telephone support, ii) psychoeducation groups, iii) a needs assessment, and the patient with psychosis was given 1-6 sessions of 0.5-1hr of psychoeducation around psychosis, treatment and management, communication, problem solving and crisis planning	Not significant		Moderate
Spandler et al., 2013[62]	Men with mental health needs (n=102)	It's a Goal: 11-week group-based cognitive behavioural programme that aimed to promote positive mental health using football metaphors (e.g. 11 matches= 11 sessions, players=service users, coaches=mental health workers)	Significant (wellbeing increased)		Moderate
Thompson et al., 2016[63]	Carers of people with hoarding disorder (n=11)	6 weekly 2 hr group psychoeducational sessions facilitated by clinical psychologists	Significant (wellbeing increased)		Moderate
**Therapy (CBT)**					
Bhutani, 2015[64]	Employees of an NHS Mental Health and Community Trust or a Local Authority (n=75)	Looking After Me Looking After You (LAMLAY): 3 3-hour or 4 2-hour weekly group CBT-based sessions	Significant (wellbeing increased)		High
Bradley et al., 2018 [65]	Young people at ultra-high risk of psychosis, identified through NHS mental health services (n=11)	SleepWell: 8 weekly sessions delivered by a clinical psychologist, using CBT techniques to improve sleep	Not tested		Moderate
Brown et al., 2019 [66]	Adolescents attending school in the London boroughs of Lambeth and Southwark who self-referred to attend a workshop to help depression and anxiety (n=155)	DISCOVER' self-referral stress management workshop programme: One day face-to-face CBT workshop delivered by clinical psychologists, followed up with a personalised 20-30 min telephone goal review and up to 2 further goal reviews within 12-weeks	Significant (wellbeing increased)	Significant (wellbeing increased)	High
Carl et al., 2020[67]	Adults with a diagnosis of generalised anxiety disorder (n=256)	Daylight: A fully automated personalised digital CBT program available on smartphones. A virtual therapist guides the participant through 4 modules (around 20 mins in length), and were suggested to use the app every day for 6 weeks	Not tested	Significant (wellbeing increased)	High
Espie et al., 2019[68]	Adults with chronic insomnia (n=1711)	Digital Insomnia therapy to Assist your Life as well as your Sleep (DIALS) study: 6 20min sessions of digital CBT delivered using the Sleepio app over 12 weeks	Significant (wellbeing increased)	Significant (wellbeing increased)	High

Reference	Participants[a]	Intervention details	Effect of intervention on wellbeing[a]		Level of confidence
			Pre- vs. Post	Compared to control	
Freeman et al., 2015[69]	People with psychosis and persecutory delusions (n=150)	6 individual 1hr sessions of CBT aiming to reduce worry	Not tested	Significant (wellbeing increased)	High
Freeman et al., 2014[70]	People with persistent persecutory delusions (n=30)	6 individual sessions of CBT over 8 weeks, provided by a clinical psychologist	Significant (wellbeing increased)	Significant (wellbeing increased)	High
Hayward et al., 2018[71]	People with a psychiatric condition and who hear voices (n=91)	Cognitive Behavioural Therapy for Psychosis (CBTp): 4 1-hr sessions of Coping Strategy Enhancement, a form of Cognitive Behavioural Therapy for Psychosis, with a clinical psychologist, counselling trainee, mental health nurse or occupational therapist	Not significant		High
Hazell et al., 2018[72]	Patients with various diagnoses from NHS mental health services who were distressed by hearing voices. (n=28)	Guided self-help cognitive-behaviour Intervention for VoicEs (GiVE): 8 weekly sessions of guided self-help CBT delivered by a clinical psychologist over a maximum of 12 weeks, based on the 'overcoming distressing voices' CBT self-help book.	Not tested	Significant (wellbeing increased)	High
Johns et al., 2019[73]	Patients with severe mental illness (n=294)	16 weekly or fortnightly 1hr CBTp therapy sessions over 6-9 months with a senior clinician	Significant (wellbeing increased)		Moderate
Loucas et al., 2020[74]	Adolescents with anxiety and/or depression (n=48)	DISCOVER: A one day group CBT workshop with individualized telephone follow-up	Not tested	Not significant	High
Miller et al., 2021[75]	Adults with moderate to severe symptoms of Generalized Anxiety Disorder (n=20)	Daylight: Digital CBT-based programme consisting of 4 10-20 minute modules and optional practice exercises	Significant (wellbeing increased)		Moderate
Powell et al., 2013[76]	Community-dwelling adults; some portfolios targets those with multiple and complex needs, young people, older people and early intervention in pregnancy and first years. (n=3070)	MoodGYM: Internet-based self-help programme teaching 5 modules of cognitive-behavioural skills; participants were encouraged to complete one module a week and sent an email reminder	Significant (wellbeing increased)	Significant (wellbeing increased)	High
Powell et al., 2020[77]	People with social anxiety symptoms not currently receiving treatment (n=0)	E-couch: 6 modules of a web-based unguided self-help intervention that used principles of cognitive behavioural therapy to help social anxiety symptoms over a 6-week period.	Not significant		High
Sheaves et al., 2018[78]	Patients admitted at acute crisis to a psychiatric hospital (n=40)	Oxford Ward sLeep Solution (OWLS): a 2-week therapy programme consisting of CBT for insomnia, sleep monitoring and light-dark exposure (minimum of 5 sessions)	Not tested	Not significant	High
The Health Foundation, 2014[79]	Patients admitted to a single male acute psychiatric inpatient ward (n=40)	Sleep treatment delivered by psychologist over 2-week period consisting of: i) cognitive behavioural techniques for insomnia; ii) sleep watches (Basis Peak) to promote patient discussion about their sleep; and iii) light therapy to stabilise circadian rhythms	Not tested	Not tested	High
Turkington et al., 2018[80]	Relatives and friends of people with psychosis (n=77)	2 (standard) or 5 (enhanced) days of a CBT-informed care in psychosis workshops	Significant (wellbeing increased)		High
Widnall et al., 2020[81]	Adults with a primary diagnosis of depression or anxiety (n=618)	Improving Access to Psychological Therapy (IAPT): 8-20 sessions of high intensity individual CBT	Significant (wellbeing increased)		High
**Therapy (other)**					
Allward et al., 2017[82]	People with mild-to-moderate dementia (n=53)	Cognitive Stimulation Therapy: 10 weekly 90-minute group sessions held at community healthcare clinics, facilitated by assistant psychologists, and includes a range of stimulating discussion, games and tasks	Not significant		High
Bacon et al., 2018[83]	Patients in secondary mental health care services (n=47)	Emotional Resources Group: 6 2.5hr emotion regulation group sessions delivered by a psychologist and a nurse	Significant (wellbeing increased)		Moderate

Reference	Participants[a]	Intervention details	Effect of intervention on wellbeing[a]		Level of confidence
			Pre- vs. Post	Compared to control	
Brown et al., 2020[84]	Healthcare staff of a Welsh health board (n=124)	Champions for Health: 12-week online self-guided Acceptance and Commitment Therapy intervention (recommended to spend 20 minutes/day, 3 days/week) as well as one of three additional interventions (i) information on behaviour change, ii) 5 premade wellbeing films, and iii) a static social norm message)	Not tested		Moderate
Cheshire et al., 2016[85]	Stressed or distressed men who were patients at a London-based GP surgery (n=102)	Atlas Men's Well-being Pilot Programme: Offered counselling (12 weekly 1-hour sessions of integrative/humanistic counselling), acupuncture (6 weekly 30min sessions) or both	Significant (wellbeing increased)		High
Collins et al., 2012[86]	University employees (n=152)	Received 1 to 19 50-min sessions from the University Counselling Service, depending on needs	Significant (wellbeing increased)	Not tested	Moderate
Dunn et al., 2019[87]	People currently experiencing a major depressive episode (n=11)	Augmented Depression Therapy (ADepT): Initial assessment of 90 mins, 15-weekly 1hr individual ADT sessions with up to 5 booster sessions with a clinical psychologist or an accredited nurse therapist	Significant (wellbeing increased)		High
Durcan et al., 2018[88]	People with history of self-harm in three prisons in North East England (n=87)	Single group session with 2 therapy dogs (activities could include sitting and petting, throwing a ball, etc)	Significant (wellbeing increased)		Moderate
Flaherty-Jones et al., 2016[89]	Older adults accessing mental health services (n=35)	Steps to Recovery: 8-weekly 1.5hr group-based therapy sessions, facilitated by two clinical mental health staff.	Significant (wellbeing increased)		High
Grajfoner et al., 2017[90]	University students from Heriot–Watt University (n=127)	Therapet: Single 20-minute group session interacting with up to 6 dogs (combined group interacting with dog only and interacting with dog and their handler)	Significant (wellbeing increased)	Significant (wellbeing increased)	High
Hartley, 2017[91]	Local people with psychological distress (n=47)	Social enterprise outreach wellbeing service that provided psychotherapy sessions from trained wellbeing workers or direct participants to local partner organisations	Significant (wellbeing increased)		High
Hemmings et al., 2021[92]	Adults diagnosed with GAD. (n=10)	Biobase ACT programme: 6 5-minute modules of a digital ACT programme completed within 2 weeks	Not significant		Moderate
Jolley et al., 2020[93]	Services users and/or their carers from community psychosis services (n=42)	4 weekly 2hr group ACTp sessions, with two additional sessions at 10 and 12 weeks	Not tested	Not significant	High
Kevern & Hill, 2017[94]	Patients attending primary care centres with mental health needs (n=107)	Chaplains for Wellbeing: Counselling sessions with trained chaplains concerning spiritual wellbeing	Significant (wellbeing increased)		Moderate
MacDonald, 2017[95]	Patients attending Regent Gardens Medical Practice with mental health needs (n=160)	Primary Care Chaplaincy: Counselling sessions with practice chaplain; session duration (up to 1hr) and frequency determined by patient need	Significant (wellbeing increased)	Not significant	Moderate
Majumdar et al., 2019[96]	Adult stroke survivors (n=53)	ACTivate Your Life after Stroke: 4 weekly 2hr didactic PowerPoint group ACT sessions	Not tested	Significant (wellbeing increased)	High
Northcott et al., 2021[97]	Adults at least 6 months post stroke with aphasia (n=32)	SFBT: Up to 6 sessions of individual solution-focused brief therapy sessions over 3 months	Not tested	Not significant	Moderate
Priebe et al., 2015[98]	People with psychosis (n=177)	DIALOG+: Monthly use of DIALOG+, a computer-assisted intervention, where patients rate their life and treatment satisfaction. Clinicians use scores to provided treatment received solution-focused therapy	Not significant	Not significant	High
Schrank et al., 2016[99]	People with psychosis (n=84)	WELLFOCUS Positive Psychotherapy: 11 weekly 90-minute sessions of WELLFOCUS Positive Psychotherapy delivered in groups by trained psychotherapists	Significant (wellbeing increased)	Not significant	High

Reference	Participants[a]	Intervention details	Effect of intervention on wellbeing[a]		Level of confidence
			Pre- vs. Post	Compared to control	
Strauss et al., 2018[100]	People with obsessive compulsive disorder (n=37)	10 2hr sessions delivered by a clinical psychologist on mindfulness-based exposure and response prevention	Not tested	Not significant	High

[a] Information on sample size and intervention effect is based on data included in analysis examining differences in total WEMWBS scores (pre-vs post-intervention in intervention group OR difference in pre- vs post-intervention or post-intervention scores between intervention and control groups)

Supplemental File S4B: Description of studies: Social interventions

Reference	Participants[a]	Intervention details	Effect of intervention on wellbeing[a]		Level of confidence
			Pre- vs. Post	Compared to control	
**Person-centred information and/or support**					
Battrick et al., 2014[101]	Individuals with multiple needs and exclusions (e.g. homelessness, substance misuse, mental health problems and offending) (n=36)	Making Every Adult Matter: Patient engages with coordinator, who ensures the best possible route through existing services (e.g. access to housing, treatment for substance misuse, or mental health assessments)	Significant (wellbeing increased)		Moderate
Beynon et al., 2020[102]	Older adults living in Bristol who are likely to be lonely and isolated (n=132)	Community Navigator: Trained 'Community Navigators' work 1:1 on a short-term basis visiting participants in their homes or via phone calls, providing free information and signposting for people experiencing isolation or loneliness, and well as to other services such as financial and safety	Significant (wellbeing increased)		High
Borschmann et al., 2013[103]	Adults with borderline personality disorder (n=49)	A 60min meeting to develop a Joint crisis plan (with key workers/family members), which was then emailed within 24 hours to the participants and key contacts.	Not significant	Not significant	High
British Red Cross, 2019[104]	Survivors of trafficking for individuals from outside of the EU (n=53)	Sustainable integration and Trafficked human beings through proactive identification and Enhanced Protection (STEP): Multiple organisations delivered complementary models of longer- term person-centred support over a 12-15 month period	Not tested		Moderate
Cheshire et al., 2018[105]	Social housing tenants in London (n=358)	Two interventions: 1) Individuals received a signposting intervention from the social housing provider which enabled the manager to identify additional needs for the participant. After the initial appointment, contact was made every 3 months for 18 months.	Not tested	Not significant	High
	as above (n=174)	2) a more-intensive intervention, 'handholding', which was delivered by an inhouse team of health and well-being support workers, who conducted baselines assessment and identified further needs of the participants and then helped participants access further services (such as by arranging travel). Participants were visited between weekly and monthly for 18-months	Significant (wellbeing increased)	Significant (wellbeing increased)	as above
Dalkin et al., 2019[106]	People attending Citizens Advice service including i) those with severe and enduring mental health issues; ii) those referred through their GP; and iii) young adults (n=191)	Intensive advice services delivered by a branch of Citizens Advice in the North East of England over 2-months to 2-years	Significant (wellbeing increased)		High
Family Mosaic, 2016[107]	Residents of Family Mosaic, a housing provider in London and the South East. (n=0)	Health Begins at Home: Intervention 1: signposted to health and wellbeing services by their neighbourhood manager;	Not significant	Not significant	High
	as above (n=0)	as above: Intervention 2: received intensive personalised support from a dedicated health and wellbeing support worker, including being accompanied to relevant local services.	Not significant		as above
Fisk, 2017[108]	Ex-seafarers in Merseyside (n=28)	Better Health for Ex-Seafarers: A series of brief interventions (no further information) delivered by a Health Project Advisor to improve participants mental and physical wellbeing and nurture self-management	Not significant		Moderate

Reference	Participants[a]	Intervention details	Effect of intervention on wellbeing[a]		Level of confidence
			Pre- vs. Post	Compared to control	
Freeman et al., 2016[109]	People with persistent persecutory delusions (n=10)	Feeling Safe: 6-month Feeling Safe programme consisting of an individual treatment meeting and a personalised 'menu' of weekly 1 hr treatment interventions for the participant to choose from (e.g. improving sleep, improving self-confidence, feeling safer)	Not significant		Moderate
Fullwood, 2018[110]	Older people with multiple long-term conditions who are at the greatest risk of avoidable hospital admissions. (n=932)	Personalised Integrated Care Programme: Coordinated, person-centred community-based approach using primary care-led multidisciplinary teams	Significant (wellbeing increased)		High
Hill-Dixon et al., 2018[111]	Homeless people living in South-West London (n=60)	Homeless Health Link: 1:1 professional and volunteer support to help participants access healthcare services; weekly group sessions to improve health and wellbeing knowledge	Not tested		Moderate
Lloyd-Evans et al., 2020[112]	Individuals with anxiety or depression using secondary mental health services (n=40)	The Community Navigators Programme: 10 1hr meetings over a 6month period with a Community Navigator who provides lessons/guidance to lonely individuals in terms of exploring their interests more and forming more social relationships with those in their community. Access to 3 group sessions is also given.	Not tested	Not tested	Moderate
Lovell et al., 2018[113]	Patients with severe mental health problems (n=604)	A shared decision-making intervention consisting of a 2-day course, 6 hours of follow up supervision and 8 hours of optional self-directed learning	Not tested	Not significant	High
Maxwell et al., 2018[114]	Patients with Long Term Health Conditions (n=67)	Patient Centred Assessment Method: Nurses assess patient using the Patient Centred Assessment Method (health and wellbeing; social environment; health literacy and communication; service co-ordination) and can then refer or signpost to other professionals or agencies	Not tested	Not tested	High
Reidy et al., 2013[115]	People with mental health problems (n=9)	Short-term social care interventions delivered by clinicians, local authorities and the third section, targeting individual's mental health problems	Not significant		Moderate
Smith et al., 2012[116]	People with mental health problems (n=25)	Advocacy in Wirral: Peer-led service which provides advice on welfare benefits, as well as advocacy on aspects such as healthcare, drug and alcohol, and hospital care	Significant (wellbeing increased)		Moderate
Thiel et al., 2013[117]	Patients with mild to moderate health problems and complex social needs (n not reported)	The Sandwell Esteem Team: Holistic co-ordinated care that includes health screening, psychiatric liaison, co-morbidities and medication management, home treatment, ambulatory pathway to home	Significant (wellbeing increased)		Moderate
Woodhead et al., 2017[118]	Adults accessing co-located welfare advice services in healthcare settings (n=901)	Welfare benefits and debt advice service co-located in healthcare settings	Not tested	Not significant	Moderate
**Parenting**					
Bjornstad et al., 2021[119]	Primary carers of children with additional needs or disability (n=92)	Healthy Parent Carers: Above (offered as 6 weekly 4-hour sessions or 12 weekly 2-hour sessions)	Not tested	Not significant	High
Borek et al., 2017[120]	White, female carers of children with additional needs or disability (n=7)	Healthy Parent Carers: 6 weekly 3-hour sessions delivered by peer facilitators consisting of health promotion education, group activities, discussions and action planning	Not tested		Moderate
Bradley et al., 2020[121]	Parents living in temporary accommodation with self- identified difficulties related to parenting a child aged 2–11years. (n=13)	Empowering Parents, Empowering Communities: Peer-led parenting programme consisting of 10 weekly sessions that comprised of facilitator demonstration, role play, visually aided discussions and review of homework tasks.	Significant (wellbeing increased)		Moderate

Reference	Participants[a]	Intervention details	Effect of intervention on wellbeing[a]		Level of confidence
			Pre- vs. Post	Compared to control	
Connect Centre University of Central Lancashire, 2021[135]	Female survivors of domestic violence and abuse (n=35)	Roadmap Programme: SafeLife: Five strands of interventions provided a 'whole family' approach for survivors, children and perpetrators	Not significant		Moderate
Cullen et al., 2013[122]	Parents (no further detail) (n=4231)	Parenting Early Intervention Programme: Interventions that made up the Parenting Early Intervention programme were all delivered to groups of parents, but the courses varied in length from five 2-hour weekly sessions to 17-weekly 2-hour sessions	Significant (wellbeing increased)		High
Department for Digital, Culture, Media & Sport, 2019[123]	Parents of children aged 2-11 who attend local EPEC hubs across England (n=348)	Being a Parent: 8 weekly 2-hr parent-delivered parenting sessions aimed at empowering participants to use positive behaviour management strategies and manage parental stress.	Significant (wellbeing increased)		Moderate
Fisher & Burchett, 2019[124]	Single parents in Wales (n=38)	Wellbeing Workshops: 6 weekly or biweekly themed sessions to equip single parents with tools and skills to promote self-care/support mental health and wellbeing, discuss issues that impact mental health and wellbeing and provide a single parents-only social network	Not tested		Moderate
Fisher & Gingell,2016[125]	Single parents in Cardiff and Newport, Wales (n=39)	Creating Connections: Programme consisted of: i) 6 weekly 4hr sessions offering skills training in goal setting, action planning and problem solving; ii) ongoing peer support; iii) support in developing individual and community goals; iv) other training and volunteering options	Significant (wellbeing increased)		Moderate
Gray et al., 2018[126]	Parents with concerns about their child(ren)'s behaviour (n=4942)	Participants in the study had received one of 3 parenting programs. 1) Incredible Years was for parents of children aged 8-13 years old and involved 18-22 weekly group sessions of 2-2.5 hours focused on teaching parents how to manage child's behavioural problems. 2)Triple P was for parents of 0-16 year olds and aimed to increase skills and confidence in handling child's behaviour through positive parenting over 8 2-hour weekly sessions. 3) STOP was an 11-week program for parents of children aged 11-16 aiming to help parents better communicate with their children	Significant (wellbeing increased)		Moderate
Harwood et al., 2021[127]	Parents experiencing difficulties in managing their infant aged 0-12 months (n=88)	Baby and Us: Peer-led early parenting programme consisting of 8 weekly 2 hr sessions with interactive learning methods to improve parental knowledge	Significant (wellbeing increased)		High
Hutchings et al., 2017[128]	Families with a child aged between 12 and 36 months living in Flying Start areas across Wales (n=89)	The Incredible Years Toddler Parenting Programme.: 12 weekly sessions using social learning theory principles to underpin the basic parent programme	Significant (wellbeing increased)	Significant (wellbeing increased)	High
Institute of Public Care Oxford Brookes University, 2020[136]	Parents/carers of children who have been exposed to domestic abuse or violence (n=154)	Opening Closed Doors (Safety, Trust and Respect (STAR) for children; Integrated Women's Support (IWS) for the female parent/carer; the Domestic Abuse Perpetrator Programme (DAPP) for the male parent/carer): Whole family interventions that can be delivered in 1:1 group format. STAR is a 10-week program for children to explore their feelings around domestic abuse, IWS is a 20 -week program for females focused on risk management, safety planning, mental health, self-esteem, parenting and support networks. DAPP is a 20-week behaviour change program using the RESPECT principles to facilitate men ending abusive behaviours towards female partners.	Significant (wellbeing increased)		Moderate
Jones et al., 2016[129]	Mothers of infants aged 2-16 weeks (n=80)	The Incredible Years Toddler Parenting Programme.: 8 weekly 2hr sessions aimed to improve parenting skills	Not significant	Not significant	High

Reference	Participants[a]	Intervention details	Effect of intervention on wellbeing[a]		Level of confidence
			Pre- vs. Post	Compared to control	
Knibbs et al., 2016[130]	Carers with a child aged 5-12 in placement (n=59)	Keeping Foster and Kinship Carers Supported: Group training programme (16 weekly 90 min sessions) aiming to improve the skills and confidence of foster and kinship carers	Not significant	Not significant	Moderate
Lindsay. et al., 2011[131]	Parents of young people aged 8-13 years demonstrating or at risk of developing behavioural difficulties receiving 1 of 3 interventions via the Parenting Early Intervention Pathfinder (n=237)	Incredible Years: 17 weekly 2hr sessions aiming to enhance effective, positive parenting	Not tested		Moderate
	as above (n=487)	Triple P Positive Parenting Program Level 4: 8 weekly 2hr sessions focusing on parental management of child behaviour and reduction of parental stress.	Not tested		as above
	as above (n=347)	Strengthening Families, Strengthening Communities: 12 weekly 3hr sessions for minority, ethnic groups targeting the development of effective parenting skills.	Not tested		as above
Lindsay & Totsika, 2017[132]	Parents of children aged 0–6 years in three local authorities in England. (n=656)	CANparent Trial: 12 universal parenting programmes aiming to improve parenting skills with varied delivery models (one of: face to face groups, face to face 1:1, blended face-to-face and online learning, only online) and length (range: weekly sessions over 2-10 weeks)	Significant (wellbeing increased)		High
Robertson et al., 2016[133]	Parents or carers of overweight or obese children (n=113)	Families for Health: 10 weekly 2.5hr sessions educating on parenting skills, social and emotional development and healthy eating	Not significant	Not significant	High
Simkiss et al., 2013[134]	Parents with children aged 2-4 years living in the catchment area of 'Flying Start' early years centres (n=245)	Family Links Nurturing Programme: 10 week universal parenting skills programme consisting of weekly 2 hr sessions	Not tested	Not significant	High
**Community and peer support**					
Chakkalackal & Kalathil, 2014[141]	Older adults with dementia living in extra care housing (n=21)	Weekly peer support groups for 6-months, which were led by an experienced facilitator. Each week there was a different focus or activity, such as creative writing and using technology	Not significant		Moderate
Get Set to Go Research Consortium, 2017[143]	Adults with mental health problems (n=798)	Get Set to Go Programme (Local delivery): Tailored peer support and 1 to 1 advice delivered by 8 local Minds across four priority regions.	Not tested	Not tested	Moderate
Jones et al., 2021[144]	Older community-dwelling adults in Bristol (n=865)	Bristol Ageing Better Projects: A city-wide programme with a wide range of initiatives to promote community involvement, participation in social activities and local decision-making, and personal support.	Significant (wellbeing increased)		Moderate
Jones et al., 2015[145]	Older adults in Plymouth (n=93)	Plymouth SeniorNet: Lessons from volunteers (~12 hours over 8 visits) on how to use the internet either in small groups or at home 1:1 sessions.	Significant (wellbeing increased)		High
Marshall et al., 2020[142]	Adults with moderate or mild aphasia caused by stroke (n=46)	Fortnightly 1.5hr social support group intervention sessions aimed to promote wellbeing and communicative success	Not significant	Not significant	High
Mental Health Foundation, 2018[140]	Older adults living in retirement or extra care housing (n=13)	Standing Together: Weekly session for 6 months that provided peer support via miscellaneous activities aimed to increase participation and aid cognitive stimulation (E.g. film quiz, discussing what to do if having a bad day)	Not significant		Moderate

Reference	Participants[a]	Intervention details	Effect of intervention on wellbeing[a]		Level of confidence
			Pre- vs. Post	Compared to control	
Moreton et al., 2018[148]	Older adults living in Birmingham who are isolated or at risk of isolation (n=434)	Ageing Better in Birmingham: Asset-based approach that aims to increase active citizenship and reduce isolation; programme elements include: i) self-organising groups of volunteers supporting isolated individuals, ii) Ageing Better hubs; iii) funding for activities; iv) service directory to help individuals find local support and activities; v) local action plans to create longer-term change; vi) supporters scheme for businesses/organisations; vii) experienced expert group who shape programme	Not tested		Moderate
Panayidou et al., 2020[139]	PhD students (n=44)	Weekly 90 minute sessions for PhD students aiming to improve wellbeing and confidence in timely PhD completion lasting for eight weeks	Not tested		Moderate
Panayiotou et al., 2020[138]	Young people in Years 7-8 that report at least one indicator of an emerging mental health difficulty (a mild or moderate emotional, behavioural, attention, or relationship difficulty) as assessed by school staff or self- nomination. Mentored by young people in Years 9-10 (n=137)	More than Mentors: Cross-age peer mentoring program (1.5 hr weekly session over a 0-12 week period), where an older pupil mentors a younger pupil with group activities, 1 to 1 mentoring working through a standardised toolkit of resources [WEMWBS assessed in mentees]	Not significant	Not tested	Moderate
	Young people in Years 9-10 who attend two-day mentor training (n=120)	as above [WEMWBS assessed in mentors]	Not significant	Not tested	as above
Parsfield et al., 2015[147]	Community residents (n not reported)	Connected Communities: Programme that works with local people to build projects that support social connections	Not tested		Moderate
Tavistock Relationship, 2019[137]	Children affected by their parent's separation and/or conflict (n=58)	1hr weekly sessions for 8-10 weeks consisting of a mutual-aid peer support group that included group work, 1-1 activities and discussions.	Significant (wellbeing increased)		High
The Health Foundation, 2015[146]	Individuals with severe Chronic Obstructive Pulmonary Disease (COPD) in Coventry (n=45)	Respiratory Innovation: Promoting Positive Life Experience: An informal weekly afternoon clinic/education session with social activities such as bingo, quizzes, singing and seated yoga over a 6-month period	Significant (wellbeing increased)		Moderate
**Social prescribing**					
British Red Cross, 2019[155]	Older people who feel loneliness or socially isolated, across the UK (n=338)	Community Connectors programme: Social prescribing service that signposts older people to range of groups and activities in local areas, including provision of emotional and practical support.	Not tested		Moderate
Bromely by Bow Centre, 2017[149]	People living with and beyond cancer (n not reported)	4 levels of social prescribing support were offered to the participants: i) telephone contact to introduce service and assess needs; ii) signposting and referral to further services; iii) face-to-face 1 hour long assessment in a community setting where participant wellbeing is explored and goals identified, actions agreed upon and referrals or signposting offered; iv) an additional 3 further 1-hour sessions if needed	Not significant		Moderate
Dayson & Leather, 2018[150]	People referred by the GP as potentially benefiting from additional socially- focused support (n=206)	Community Connector finds out what the participant is interested in, identify what services and activities are available locally that fit those interests, and help the participants access them	Not tested		Moderate

Reference	Participants[a]	Intervention details	Effect of intervention on wellbeing[a]		Level of confidence
			Pre- vs. Post	Compared to control	
Giebel et al., 2021[151]	People living with dementia and family carers (n=25)	Happy and Healthy: Socially prescribed weekly 60min classes involving light physical activity, mindfulness and games, over a 6-month period	Significant (wellbeing increased)		Moderate
Morton et al., 2015[154]	Adults with mild to moderate mental health problems (n=136)	Consultation with service user and social prescribing of 1-3 sessions of 6 courses (meditation, painting, photography, jewellery, arts & crafts, pottery)	Significant (wellbeing increased)		Moderate
Pescheny et al., 2021[152]	Primary care patients with psychosocial needs referred to social prescribing scheme (n=63)	Patients were referred by link worker to 12 free sessions of the Luton Social Prescribing Scheme which involved physical activities, social activities and creative activities.	Significant (wellbeing increased)		Moderate
Southmead Development Trust et al., 2018[153]	Patients attending 6 GP practices in Bristol (n=93)	Community Webs: Project coordinator and link-workers based in GP practices use social prescribing to enable and equip patients to access social activities and non-medical support services available in their local community	Significant (wellbeing increased)		Moderate

[a] Information on sample size and intervention effect is based on data included in analysis examining differences in total WEMWBS scores (pre-vs post-intervention in intervention group OR difference in pre- vs post-intervention or post-intervention scores between intervention and control groups)

Supplemental File S4C: Description of studies: Arts, culture, and environment

Reference	Participants[a]	Intervention details	Effect of intervention on wellbeing[a]		Level of confidence
			Pre- vs. Post	Compared to control	
**Art**					
Barker & Winship, 2016[156]	People recovering from substance misuse problems (n=4)	Laughing Matters: Comedy workshops led by a professional comedian over 4 weeks, where participants developed, wrote and performed a stand-up comedy routine	Not significant		Moderate
Clift et al., 2017[157]	Adults with mental health issues (n=25)	The West Kent and Medway Project: Weekly singing groups in community venues for 6 months	Significant (wellbeing increased)		High
Crone et al., 2017[158]	Individuals with a current or previous diagnosis of cancer (n=17)	Flourish programme: 8 weekly art course sessions in a variety of mediums	Significant (wellbeing increased)		Moderate
Crone et al., 2018[159]	Individuals with: i) anxiety, depression or stress; ii) low self-esteem, confidence or overall wellbeing; iii) and chronic illness or pain (n=1258)	Art Lift: Weekly art class for 10 weeks taught on a single topic (e.g., poetry, ceramics, drawing, mosaic, painting)	Significant (wellbeing increased)	Not significant	Moderate
Efstathopoulou & Bungay, 2021[160]	Adolescents at risk of behavioural or emotional problems in the East of England (n=65)	Arts on Prescription (AoP): 10-weekly 2hr visual art workshops delivered in schools	Significant (wellbeing increased)		High
Fancourt et al., 2015[161]	Adults with affective disorders accessing mental health services (n=31)	6-weekly 70-minute group drumming sessions	Significant (wellbeing increased)		High
Fancourt et al., 2016[162]	Patients using mental health services (n=45)	10 weekly 90-minute group drumming sessions	Significant (wellbeing increased)	Significant (wellbeing increased)	High
Fancourt et al., 2019[163]	Family carers of people with cancer (n=62)	12 weekly 90minute choir sessions	Significant (wellbeing increased)	Significant (wellbeing increased)	High
Glenister, 2017[164]	Youth in challenging circumstances referred from mental health services, social services or education sources (separate sample to 164) (n=35)	Noise Solution: 10x weekly 2 hr sessions pairing youth with informal music producers for one-to- one project-based tutoring, centred around the use of music technology	Significant (wellbeing increased)		High
Margrove, 2015[165]	Students studying at Anglia Ruskin University (n=7)	Open Arts: Weekly 2hr sessions for 12 weeks consisting of a variety of visual arts classes	Not significant		Moderate
Margrove et al., 2013[166]	Individuals with mental health needs (n=58)	Open Arts: Weekly 2hr sessions for 12 weeks consisting of a variety of visual arts classes	Significant (wellbeing increased)	Significant (wellbeing increased)	Moderate
Nevay et al., 2019[167]	Community-dwelling adult females (n=15)	Crafting connections for wellbeing using e- textiles: Single textile-based workshop to construct 3D interactive birds	Significant (wellbeing increased)		Moderate
Poerio & Totterdell, 2020[168]	Older community-dwelling adults (n=94)	Single audiobook listened to via MP3 player over a 4-week period	Not significant	Not significant	High
Ribbans & Glenister, 2019[169]	Youth in challenging circumstances referred from mental health services, social services or education sources (separate sample to 169) (n not reported)	Noise Solution: 10x weekly 2 hr sessions pairing youth with informal music producers for one-to- one project-based tutoring, centred around the use of music technology	Significant (wellbeing increased)		Moderate

Reference	Participants[a]	Intervention details	Effect of intervention on wellbeing[a]		Level of confidence
			Pre- vs. Post	Compared to control	
Secker et al., 2011[170]	People at risk of mental ill health and isolation as well as those in recovery from mental health problems. (n=107)	Open Arts: One of 29 introductory arts courses lasting 10-15 weeks and involved using a variety of media	Significant (wellbeing increased)		High
Sumner & Hughes, 2021[171]	Patients/service users, their families or carers who are experiencing mental health challenges (n=33)	See with Fresh Eyes: 8 in-person half-day sessions on mindful photography techniques; shifted to 4 weekly 2 hr online sessions during the pandemic	Not tested		Moderate
Tribe et al., 2021[172]	People with low mood and anxiety (n=55)	Creativity in Mind: Participants were part of a WhatsApp group for 30 days, facilitated by a trained artist, where they took part and shared a daily creative challenge (e.g. draw your mood, create a balance sculpture, etc.)	Significant (wellbeing increased)		High
van de Venter & Buller, 2014[173]	People with mild-to-moderate mental health problems (n=44)	Arts on Referral: 20 week regular art sessions incorporating a variety of arts activities (e.g. painting, textiles, music, photography and film)	Significant (wellbeing increased)		High
Wilson et al., 2017[174]	Individuals with mental health needs (n=74)	Open Arts: Weekly 2hr sessions for 12 weeks consisting of a variety of visual arts or drama classes or a 6 week percussion course	Significant (wellbeing increased)		Moderate
**Culture**					
Ecorys, 2017[177]	Residents of villages and towns in North East England (n not reported)	Bait: Programme of activities aimed at increasing participation in arts and culture. Part of Creative People and Places (CPP) programmes funded by Arts Council England.	Not tested		Low
Heaslip & Darwill, 2018[176]	Individuals with long-term mental health needs (n not reported)	Human Henge: 10 half day sessions of participant-led activities coupled with experts, carers, support workers, and contributors from a range of cultures who together explored prehistoric landscapes in the Stonehenge and Avebury World Heritage Site	Not tested		Moderate
Khan et al., 2017[175]	Young African Caribbean men in Birmingham (n=70)	Up My Streets: Three projects: 1) workshops and activities for young men to explore their culture and heritage and lead a local social media campaign to promote resilience, empathy, and aspiration; 2) a homelessness charity organised workshops and visits on Black History, personal development and resilience; 3) a theatre company offered drama workshops	Significant (wellbeing increased)		Moderate
**Environment**					
Beishon et al., 2016[178]	Community residents, often from deprived backgrounds or with physical/mental health problems, can self-refer or are referred by local health, social care and voluntary sector partners (n not reported)	Green Gym: Ongoing weekly 3-4 hour activity at a local community facility (e.g. park) consisting of a warm-up, cool-down, and gardening and land management activities	Not tested		Moderate
Chiumento et al., 2018[179]	Children experiencing behavioural, emotional, and social difficulties (n=31)	A Haven of Green Space: In monthly 2-hour sessions over 6-months, the children designed a green space facilitated by two horticulturists and a Child and Adolescent Mental Health Service psychotherapist	Not significant		Moderate

Reference	Participants[a]	Intervention details	Effect of intervention on wellbeing[a]		Level of confidence
			Pre- vs. Post	Compared to control	
Farrier et al., 2019[180]	Male prisoners in 12 prisons in North East England (n=135)	Greener On the Outside of Prisons (GOOP): Depending on prison type and prisoner category, individuals participated in various horticultural and environmentally focused projects and training courses	Not tested		Low
Kearns et al., 2020[181]	Adults living in areas undergoing regeneration in Glasgow. (n=1398)	A regeneration programme carried out across 15 communities in Glasgow. Intervention area types included: i) regeneration area; ii) Wider surrounding area; iii) High-rise Housing Improvement Area; iv) Low-rise Housing Improvement Area	Not significant		Moderate
Maund et al., 2019[182]	Individuals diagnosed with anxiety and/or depression (n=18)	The Wetland NBI Design: 6 weekly 2hr sessions engaging participants with nature with different activities each week.	Significant (wellbeing increased)		Moderate
Sumner et al., 2020[183]	Outpatients with cardiovascular health conditions currently receiving care (n=19)	Nature on Prescription: 8 week course (~5 hours/week) of a variety of nature-based activities, centring walks and wild outdoors programmes (including practical conservation), incorporating education regarding cardiac health.	Significant (wellbeing increased)		Moderate
Wilson et al., 2011[184]	Patients referred from secondary or tertiary mental health services in the Greater Glasgow and Clyde area (Scotland) (n=77)	Branching Out: 12-weeks of ~3 hrs of group ecotherapy (e.g. conservation, bushcraft, environmental art, construction, exercise) in outdoor woodland settings.	Not significant		High

[a] Information on sample size and intervention effect is based on data included in analysis examining differences in total WEMWBS scores (pre-vs post-intervention in intervention group OR difference in pre- vs post-intervention or post-intervention scores between intervention and control groups)

Supplemental File S4D: Description of studies: Physical health promotion

Reference	Participants[a]	Intervention details	Effect of intervention on wellbeing[a]		Level of confidence
			Pre- vs. Post	Compared to control	
**Physical activity**					
Connolly et al., 2020[185]	Premenopausal women physically inactive women (n=24)	Participants completed a 12-week physical activity programme, delivered via DVD, where they completed 15-minutes of exercise 3 times a week. The exercise consisted of low-, moderate-, and high-in10sity physical activity.	Significant (wellbeing increased)	Not significant	High
Corepal et al., 2019[186]	Adolescents from five schools in Belfast, Northern Ireland (n=213)	The StepSmart Challenge: 22-week intervention using gamification strategies (schools participated in a pedometer-based competition to encourage and support physical activity behaviour change)	Not tested	Not tested	High
France et al., 2016 [187]	Individuals affected by cancer and other long-term health conditions as well as those from specific health inequality groups (e.g. older adults, black and minority ethnic communities, low income). (n=232)	Walking for Health: England-wide network of schemes that delivers free, group-walks that are open to all and 10-90min in duration at least once monthly	Significant (wellbeing increased)		High
Gray et al., 2020[188]	Adults living in care homes and supported housing environments (n=70)	Cycling Without Age: Participants go out on specially designed trishaws piloted by trained volunteers, each ride lasted for 40-60 minutes	Significant (wellbeing increased)	Significant (wellbeing increased)	High
Hadley et al., 2020[189]	Patients with Parkinson's disease and their partners or carers (n=40)	Participants (and some of their carers/partners) completed a single 30-40min dance class specifically designed for people with Parkinson's.	Significant (wellbeing increased)		Moderate
Harris et al., 2018[190]	Residents of Stranraer, Scotland (no further description) (n=167)	Beat the Street: Over a 6-week period, individuals accrued points and prizes by scanning a card at consecutive electronic boxes (at half-mile intervals) within an hour. This was followed by 7-months of directing participants into suitable physical activity opportunities.	Significant (wellbeing increased)		High
Henderson et al., 2014[191]	Primarily individuals referred from primary care/mental health providers and voluntary sector organisations (e.g. local Mind groups) (n=96)	Imagine Your Goals: Football-based exercise programme that ran during the 2010-11 season (frequency not stated)	Not significant		High
Hunter et al., 2018[192]	Employees of public sector organisations in Northern Ireland (n=853)	The Physical Activity Loyalty Scheme (PAL): Over a 6-month period, individuals accrued points and prizes by carrying their PAL key fob within 25m of WIFI beacons placed within 2km of their work place.	Not tested	Significant (wellbeing increased)	High
Kay et al., 2022[193]	Navy, army and RAF who are either wounded, injured or sick with mental or physical health-related illness (separate sample to 196) (n=759)	Battle Back Multi-Activity Course: 5 day residential course consisting of multi-sport activities and self-management education aimed to encourage and equip individuals to improve their mental and physical health	Significant (wellbeing increased)		Moderate
Lewis et al., 2017[194]	Older harder-to-reach men in North West England (n=80)	The Active Rovers: Mixed exercise programmes (e.g. football, yoga, Tai Chi) delivered on a weekly basis at Prenton Park, the home of Tranmere Rovers FC	Significant (wellbeing increased)		Moderate

Reference	Participants[a]	Intervention details	Effect of intervention on wellbeing[a]		Level of confidence
			Pre- vs. Post	Compared to control	
Malcolm et al., 2013[195]	Participants referred by mental health services, social services, local mental health organisations or an educational authority (n=2663)	MIND and Rethink Mental Illness Exercise Project: 28 community-based exercise projects around England; lasting 3-12 months, each project consisted of weekly activities (e.g. gardening and conservation, gym classes, walking)	Significant (wellbeing increased)		Moderate
Peacock et al., 2019[196]	Navy, army and RAF who are either wounded, injured or sick with mental or physical health-related illness (separate sample to 193) (n=971)	Battle Back Multi-Activity Course: 5 day residential course consisting of multi-sport activities and self-management education aimed to encourage and equip individuals to improve their mental and physical health	Significant (wellbeing increased)		Moderate
Tew et al., 2017[197]	Physically inactive older adults (n=47)	10x 75-minute Yoga sessions over 12-weeks, delivered by trained Yoga teachers	Not tested	Significant (wellbeing increased)	High
Walker et al., 2021[198]	People with obesity, type 2 diabetes or pre-diabetes (n=17)	Participants attended a Zoom-delivered, online weight loss and health promotion intervention, delivered in 6x 90-minute sessions over 10-weeks	Significant (wellbeing increased)		Moderate
**Health promotion (diet or mixed)**					
Newbury-Birch et al., 2014[199]	Year 10 pupils in North East London (n=107)	Participants received a brief alcohol screening intervention, delivered by school learning mentor. Two interventions were tested: 1) Participants received 30-minute brief interactive session involving structured advice about alcohol harms and motivational interviewing	Not tested	Not tested	High
	as above (n=75)	2) included everything delivered in intervention 1 plus a 60-minute session involving family members	Not tested	Not tested	as above
Giles et al., 2019[200]	Year 10 pupils who screened positively on a single alcohol screening question (n=401)	30-minute one-to-one structured brief intervention with a trained learning mentor and an alcohol leaflet.	Not tested	Not significant	Moderate
Johnson et al., 2017[201]	Local community-dwelling individuals recruited from media publicity, GP 'referral' and posters (n=481)	One Body One Life: 12 weekly 90-min sessions consisting of 45min of exercise and a 45-min workshop on healthy eating	Significant (wellbeing increased)		Moderate
Callaghan et al., 2019[202]	People under community supervision in the criminal justice system (n=120)	STRENGTHEN: Person-centred health trainer support in one-to-one sessions for up to 14 weeks, either in person or via telephone. Health trainers aimed to empower participants to make healthy lifestyle changes (particularly in alcohol use, smoking, diet and physical activity)	Not tested	Not tested	High

[a] Information on sample size and intervention effect is based on data included in analysis examining differences in total WEMWBS scores (pre-vs post-intervention in intervention group OR difference in pre- vs post-intervention or post-intervention scores between intervention and control groups)

Supplemental File S4E: Description of studies: Other

Reference	Participants[a]	Intervention details	Effect of intervention on wellbeing[a]		Level of confidence
			Pre- vs. Post	Compared to control	
**Funding**					
CLES Consulting & New Economics Foundation, 2013[203]	Community-dwelling adults; some portfolios targets those with multiple and complex needs, young people, older people and early intervention in pregnancy and first years. (n=305)	Wellbeing Programme and Changing Spaces Programme: Part of 17 Big Lottery wellbeing programme funding portfolios of programmes across England that aimed to create healthier lifestyles and improve community wellbeing via physical activity, healthy eating, mental wellbeing	Significant (wellbeing increased)		High
Scott et al., 2014[204]	Community-dwelling adults; some portfolios targets those with multiple and complex needs, young people, older people and early intervention in pregnancy and first years. (n not reported)	Wellbeing 2: Funding to 14 portfolios across England that aimed to create healthier lifestyles and improve community wellbeing via physical activity, healthy eating, mental wellbeing	Not tested		Moderate
Age UK, 2013[205]	Older adults (n=71)	Fit as a Fiddle: 2 national projects and 24 regional projects, delivered by over 200 organisations aiming to broaden and increase the opportunities for older people to undertake physical activities and improve their eating habits, contributing to an overall improvement in mental health.	Significant (wellbeing increased)		Moderate
Wigfield et al., 2015[206]	Older adults living with at least one long term health condition (n=840)	Fit for the Future: Part of the Fit as a Fiddle Funding portfolio and delivered by 11 Age UK partners. Participants are matched with a volunteer who develops a tailored personal plan and activities to meet their needs.	Significant (wellbeing increased)		High
Scanlon et al., 2021[207]	Young people attending youth services in six regions across England (no individual level description) (n=813)	Youth Investment Fund grants: Youth Investment Fund investment across 6 regions of England to youth organisations that provide frontline, open access youth services in the targeted communities across England	Significant (wellbeing increased)	Significant (wellbeing increased)	Moderate
Day et al., 2020[208]	Up to 100 schools, colleges and Children and Young People's Community Organisations (CYPCOs) with children and young people as the main intervention target (n=322)	Peer Support for Mental Health Pilots: Funding programme delivered by up to 100 schools, colleges and Children and Young People's Community Organisations (CYPCOs) to set up and deliver bespoke peer support interventions	Not significant	Not significant	Moderate
Ministry of Housing, Communities and Local Government, 2019[209]	Parent/carer of a family that is experiencing one of following problems: worklessness, poor school attendance, mental and physical health problems, crime and anti-social behaviour, domestic violence and abuse or children in need of help and protection (n not reported)	Troubled Families Programme: Funding (£920 million) to local authority areas to deliver programmes focused on a high-level theory of change including: i) whole family approach; ii) multi-agencies involved; iii) early intervention; iv) focusing on outcomes and data	Not significant		Moderate
**Targeted medical**					

Reference	Participants[a]	Intervention details	Effect of intervention on wellbeing[a]		Level of confidence
			Pre- vs. Post	Compared to control	
Acton et al., 2016[210]	Individuals with low vision who are eligible for the Sight Cymru visual rehabilitation service (n=67)	1 to 11 home visits to assess needs of individual with low vision, and offer training and support to improve visual function outcomes	Not significant	Not tested	High
Basu et al., 2018[211]	Parents/carers/therapists of infants with perinatal stroke or unilateral haemorrhagic parenchymal infarction (n=20)	Early Therapy in Perinatal Stroke (eTIPS): Parents were taught to change the environment around the infant (for first 6-months of life) to promote opportunities for active use and stimulation of the potentially affected side of the body	Not significant		Moderate
Elnazer et al., 2021[212]	People with a primary diagnosis of an anxiety disorder (n=27)	Celecoxib augmentation (typically used to treat pain) for 6-weeks alongside current medication	Significant (wellbeing increased)	Not significant	Moderate
Evans et al., 2018[213]	Older euthymic adults with and without cardiovascular risk factors and healthy younger adults (n=120)	Transcranial random noise stimulation (tRNS): Participants attended 2 sessions between 2 and 14 days apart where they received transcranial random noise stimulation or sham (control) intervention	Not significant	Not significant	Moderate
Osborn et al., 2018[214]	Patients with severe mental illness and raised cholesterol (n=327)	The Primrose Intervention: Weekly or fortnightly appointments to agree goals to lower cardiovascular disease risk (e.g. Improving diet, physical activity, reduced alcohol, quitting smoking, adhering to statins) with a nurse for 6 months	Not tested	Not significant	High
Steel et al., 2020[215]	Adults with a clinical diagnosis of schizophrenia and at least a mild level of depression (n=100)	Positive Memory Training (PoMeT): 8-12 individual sessions of positive memory training over a 3-month period	Not significant	Not significant	High
Stuttard et al., 2021[216]	Individuals with severe and profound hearing loss who were first time applicants for a hearing dog (n=112)	Hearing Dogs for Deaf People: Participants received a hearing dog earlier than the control group (immediate vs 6-36 months)	Not tested	Significant (wellbeing increased)	High
**Other (professional training)**					
Slade et al., 2015[217]	Patients with psychosis who were receiving care from a community-based adult mental health team (n=403)	REFOCUS: 1 year behavioural and interpersonal intervention delivered to community mental health teams to change the way they deliver care. Staff participating in intervention were offered 12hr of training in personal recovery, 16hr of training in recovery coaching, 6 externally facilitated team-manger reflection groups, 6 internal team reflections and the use of a reflective practice tool	Not significant	Not significant	High
Webber et al., 2019[218]	People with a mental health condition or a learning disability (n=116)	Connecting People Intervention: Participants' healthcare practitioners (e.g. social care workers, nurses, psychologists, occupational therapists, psychiatrists) received a 2-day training course on how to more effectively help participants develop social networks	Significant (wellbeing increased)		High
Stansfeld et al., 2015[219]	Employees and managers of an NHS Mental Health Trust (n=284)	Managing Employee Pressure at Work: An e-learning health promotion programme focusing on the 6 management standards domains (change, control, demands, support, relationship, role) delivered in 6 modules over a 3 month period	Not significant	Not significant	High
**Mental health promotion (recovery college)**					
Ebrahim et al., 2018[220]	Students at a Recovery College aiming to enhance independence in those with mental health problems in Northern England (n=56)	Education-based mental health resources, utilising practitioner and lived experience expertise through courses at a Recovery College	Significant (wellbeing increased)		Moderate

Reference	Participants[a]	Intervention details	Effect of intervention on wellbeing[a]		Level of confidence
			Pre- vs. Post	Compared to control	
Mental Health Foundation, 2016[221]	Individuals with complex mental health needs (n=36)	Progression Together: Personalised residential service consisting of three stages over 2.5 year period that progress from intensive phase of recovery and support to independent living	Significant (wellbeing increased)		Moderate
Harrison et al., 2017[222]	Individuals with substance misuse problems (n=10)	Recovery central programmes that supports the development of abstinence-based recovery communities in four areas (Birmingham, Gloucester, London, Durham)<br>Give It Up	Not tested		Moderate
	as above (n=12)	The Hub: as above	Not tested		as above
	as above (n=20)	Progression and Choices: as above	Not tested		as above
	as above (n=11)	Clean & Sober Living: as above	Not tested		as above
Lamb et al., 2021[223]	Individual in mental health crises (n=431)	Acute day unit (4 different services across 4 trusts)	Not tested		Moderate
	as above (n=241)	Crisis resource team (4 different services across 4 trusts)	Not tested		as above
Wilson et al., 2019[224]	Students attending the South East Essex Recovery College (n=25)	South East Essex Recovery College: Uses a psychoeducational/vocational training and social/peer support approach to support people through mental health recovery and support transition from dependency to self-management	Significant (wellbeing increased)		Moderate
**Other**					
Collis & Eggers, 2020[228]	Students in the Faculty of Economics and Business at a large European university (n=122)	9 week period of restricted social media use via a mobile app that blocks Facebook, Instagram and Snapchat after 10minutes of daily use	Not tested	Not significant	High
Connect Centre University of Central Lancashire, 2021[135]	Female survivors of domestic violence and abuse (n=77)	Roadmap Programme: VOICES: Framework, training and coaching for frontline domestic and violent abuse practitioners. Compared to normal practices, it provides a new assessment framework, training and planning tools that have a gender-neutral, strengths-based, needs-led, trauma informed approach.	Not significant		Moderate
Elphick et al., 2019[226]	Parents of children with i) sleep problems and ii) one of Attention Deficit Hyperactivity Disorder or Looked After/Adopted children (n=56)	Sleep education and behavioural sleep programme delivered via a 3hr workshop or a 1:1 clinical visit, followed by an individual consultation and development of an individualised sleep programme	Significant (wellbeing increased)		High
Evans et al., 2019[229]	People with a dementia diagnosis living at home (n=77)	Dementia Dwelling Grant (DDG): Provision of small-scale aids and home adaptations including key locators and clock, touch bedside lights and bath mats	Not significant		Moderate
Gensler & the Helen Hamlyn Centre for Design, 2016[227]	Employees in a London-based organisation (n=27)	The Participatory Design Project: 3 employee teams were supported to create and test design solutions for their workspaces through a co-design workshop and design installations in their offices.	Significant (wellbeing increased)	Not tested	Moderate
Naruse et al., 2019[225]	Health but stressed couples (n=42)	3 consecutive weekly 1 hour couples massage classes	Significant (wellbeing increased)	Not significant	High

[a] Information on sample size and intervention effect is based on data included in analysis examining differences in total WEMWBS scores (pre-vs post-intervention in intervention group OR difference in pre- vs post-intervention or post-intervention scores between intervention and control groups)

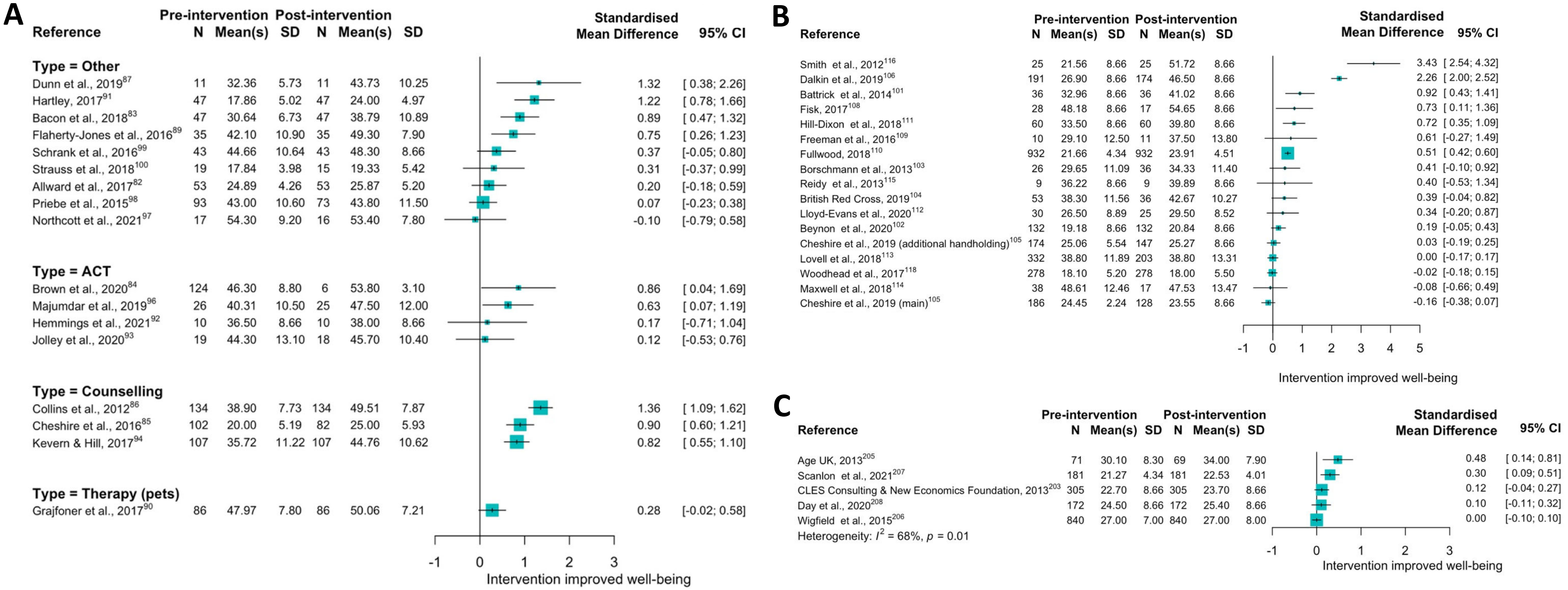

Supplemental **File S5.** Forest plot indicating change in WEMWBS score from pre to post intervention for A. Theme 1: Other therapies; B. Theme 2: Person-centred advice/support; and C. Theme 5: Funding. Change indicated by standardised mean difference.
